# Association of Social Determinants of Health and Vaccinations With Child Mental Health During the COVID-19 Pandemic in the US

**DOI:** 10.1001/jamapsychiatry.2022.0818

**Published:** 2022-04-27

**Authors:** Yunyu Xiao, Paul Siu-Fai Yip, Jyotishman Pathak, J. John Mann

**Affiliations:** 1 Department of Population Health Sciences, Weill Cornell Medicine, New York–Presbyterian, New York; 2 Hong Kong Jockey Club Centre for Suicide Research and Prevention, The University of Hong Kong, Hong Kong; 3 Department of Social Work and Social Administration, The University of Hong Kong, Hong Kong; 4 Department of Psychiatry, Columbia University Irving Medical Center, Columbia University, New York, New York; 5 Department of Radiology, Columbia University Irving Medical Center, Columbia University, New York, New York; 6 Division of Molecular Imaging and Neuropathology, New York State Psychiatric Institute, New York

## Abstract

**Importance:**

The COVID-19 pandemic disproportionately affected mental health in socioeconomically disadvantaged children in the US. However, little is known about the relationship of preexisting and time-varying social determinants of health (SDoH) at individual and structural levels, vaccination eligibility/rates, and the racial and ethnic differences to trajectories of child mental health during the COVID-19 pandemic.

**Objective:**

To estimate the association of trajectories of child mental health to multilevel SDoH and vaccination eligibility/rates.

**Design, Setting, and Participants:**

This prospective longitudinal cohort study, conducted from May 16, 2020, to March 2, 2021, integrated structural-level, pandemic-related data with the Adolescent Brain Cognitive Development (ABCD) cohort data (release 4.0). The ABCD study recruited 11 878 children (baseline) and conducted 6 COVID-19 rapid response surveys across 21 US sites (in 17 states) from May 16, 2020, to March 2, 2021.

**Exposures:**

Preexisting individual (eg, household income) and structural (area deprivation) SDoH and time-varying individual (eg, food insecurity, unemployment) and structural (eg, social distancing, vaccination eligibility/rates) SDoH.

**Main Outcomes and Measures:**

Perceived Stress Scale, the National Institutes of Health–Toolbox emotion measures, and COVID-19–related worry.

**Results:**

The longitudinal sample included 8493 children (mean [SD] age, 9.93 [0.63] years; 5011 girls [47.89%]; 245 Asian [2.34%], 1213 Black [11.59%], 2029 Hispanic [19.39%], 5851 White [55.93%], and 1124 children of other/multiracial ethnicity [10.74%]). Trajectories of stress, sadness, and COVID-19–related worry decreased after adult vaccination rollout. Compared with younger children, boys, White children, or those living with married parents, those who reported greater perceived stress included older children aged 12 to 15 years (β = 0.26; 95% CI, 0.12-0.41; *P* < .001); girls (β = 0.75; 95% CI, 0.61-0.89; *P* < .001); Hispanic children (β = 0.24; 95% CI, 0.01-0.47; *P* = .04); children living with separated parents (β = 0.50; 95% CI, 0.03-0.96; *P* = .04); children experiencing disrupted medical health care access (β = 0.19; 95% CI, 0.01-0.36; *P* = .04); children living in economically deprived neighborhoods (β = 0.28; 95% CI, 0.05-0.51; *P* = .02); children living in areas with more full-time working-class adults who were unable to social distance (β = 1.35; 95% CI, 0.13-2.67; *P* = .04); and children living in states with fewer fully vaccinated adults (β = 0.59; 95% CI, 0.16-1.02; *P* = .007). COVID-19 pandemic–related worry was higher among Asian children (β = 0.22; 95% CI, 0.08-0.37; *P* = .003), Black children (β = 0.33; 95% CI, 0.22-0.43; *P* < .001), children of other/multiracial ethnicity (β = 0.17; 95% CI, 0.09-0.25; *P* < .001), and children with disrupted medical health care (β = 0.15; 95% CI, 0.09-0.21) and disrupted mental health treatment (β = 0.11; 95% CI, 0.06-0.16). Inability to afford food was associated with increased sadness (β = 1.50; 95% CI, 0.06-2.93; *P* = .04). States with later vaccination eligibility dates for all adults were associated with greater COVID-19–related worry (β = 0.16; 95% CI, 0.01-0.31; *P* = .03) and decreased positive affect (β = −1.78; 95% CI, −3.39 to −0.18; *P* = .03) among children.

**Conclusions and Relevance:**

Results of this study suggest a disproportionately adverse association of the COVID-19 pandemic with child mental health among racial and ethnic minority groups, which may be improved by addressing modifiable individual (food insecurity, unemployment, health services, parental supervision) and structural (area deprivation, job protection, vaccination) SDoH.

## Key Points

**Question:**

To what extent are individual and structural social determinants of health (SDoH) and vaccinations associated with child mental health during the COVID-19 pandemic?

**Findings:**

In this cohort study of 8493 US children, pandemic-related food insecurity, parental unemployment, disrupted mental health treatment, living in neighborhoods with higher shares of adults working full-time, and living in states lagging in vaccination rates were associated with increased trajectories of perceived stress, sadness, and COVID-19–related worry. Associations between SDoH and these mental health outcomes were more common among Asian, Black, and Hispanic children more than White children.

**Meaning:**

Supporting children’s mental health requires multifaceted policies that address SDoH and structural barriers to food, health services, employment protection, and vaccination.

## Teaser

This cohort study investigates the association of individual and structural social determinants of health and vaccinations with child mental health during the COVID-19 pandemic in the US.

## Introduction

The COVID-19 pandemic elevated mental distress in young and older adults moderated by sociodemographic disparities.^1,2,3,4^ Few studies have examined the long-term relationship of social determinants of health (SDoH), defined as conditions in which people “…are born, grow, live, work, and age,”^5^^p(76)^ during the pandemic to children’s mental health trajectories.^6,7,8,9^ Multiplicative pandemic-related stressors and trauma from parental loss, school closures, financial uncertainties, and health care disruptions were experienced by children potentially affecting their mental health.^10,11,12,13,14^ Children aged 5 to 11 years were the last to receive vaccines (November 2021),^15^ and children younger than 5 years are still waiting for vaccine protection, lengthening their uncertainties.^8,13,16^ The syndemic of COVID-19, structural racism, and SDoH may interact with and affect mental health disparities in children during COVID-19 and in future public health emergencies.^17,18,19^

**Figure 1.**
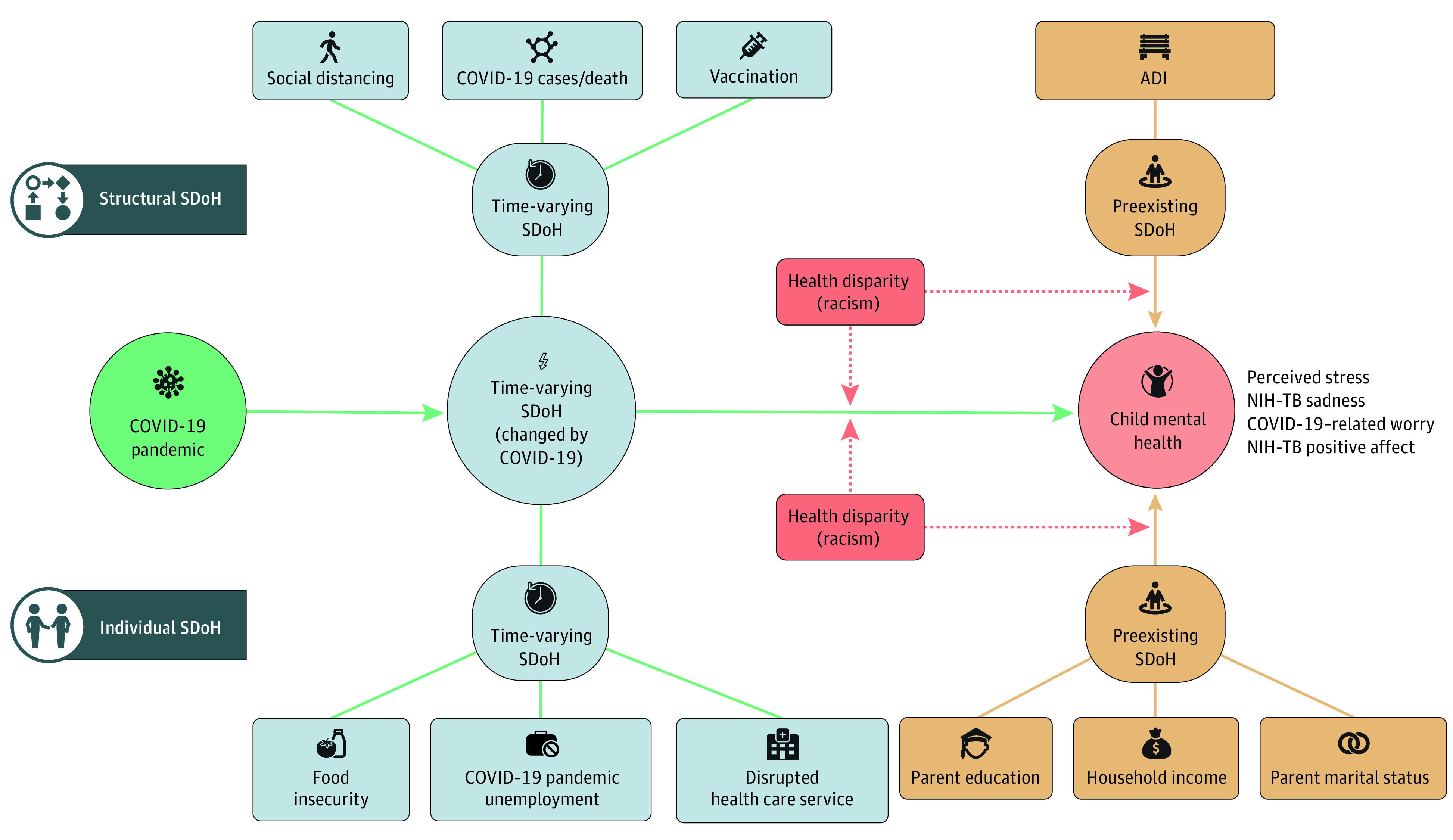
Conceptual Framework Conceptual framework of our study is adapted from Bernardini and colleagues.^19^ Reading from left to right for the time dimension (longitudinal trajectories of short- and long-term COVID-19 outcomes), and top to bottom for the space dimension (multilevel nature of social determinants of health [SDoH]). The dark blue box denotes the levels of SDoH (individual, structural). The green circle denotes the influences of the COVID-19 pandemic. The pink circle denotes the child mental health outcomes. Yellow boxes denote the preexisting SDoH. Light blue boxes and circles denote the time-varying SDoH. The red box denotes the demographic characteristics. Green arrows represent time-varying SDoH as direct risks and mediators. Solid yellow arrows denote the direct effect of preexisting SDoH. Dashed red arrows are the possible moderating association of preexisting SDoH that was not tested in the current study (eTable 2 in the Supplement). ADI indicates Area Deprivation Index (eTable 1 in the Supplement).

SDoH acts as direct risks, moderators, and mediators of mental health disparities at the individual (eg, income) and structural (eg, area deprivation) level (Figure 1).^18,19,20,21^ Preexisting SDoH underlie long-standing socioeconomic disadvantages.^18,22^ Time-varying SDoH highlight pandemic-related food insecurity, loss of parental or teacher supervision (eg, when full-time working parents were unable to work from home), disrupted health care services, and financial hardship.^2,3,22,23,24^ Children may experience more mental health and social changes from the COVID-19 pandemic than adults owing to multidimensional SDoH.^10,14,25^ Addressing SDoH can inform multilevel, equitable policy responses to protect child mental health.^19,26^

SDoH may affect children from racial and ethnic minority groups differently owing to structural racism in school and residential segregation.^10,11,25,27^ Racial differences in adult COVID-19 mortality and positivity are reported in neighborhoods with the same socioeconomic deprivation.^22,28^ Belonging to a particular race is not an SDoH. Instead, racial status is a proxy of structural racism that forms the social milieu of mental health disparities.^29^ Examining the intersection between SDoH, race, and ethnicity can guide meaningful policy solutions to health disparities.

COVID-19–related mental health studies have, to date, focused on adults^3,10,27,30^ and neglected children and social-geographic disparities. Few studies (none in children, to our knowledge) used a longitudinal study design measuring individual and structural, time-varying SDoH factors.^8,31,32^ Distilling heterogeneity in the associations between prepandemic and postpandemic SDoH risks and short- and long-term child mental health trajectories, provides critical information to design tailored and more effective prevention, treatment, and policies to mitigate race and ethnicity disparities.^32^

This study examined the association of preexisting and time-varying SDoH factors, at individual and structural levels, with mental health trajectories among US children, surveyed 6 times between May 2020 and March 2021. We further examined racial and ethnic disparities in SDoH and mental health.

## Methods

### Study Design, Setting, and Population

**Figure 2.**
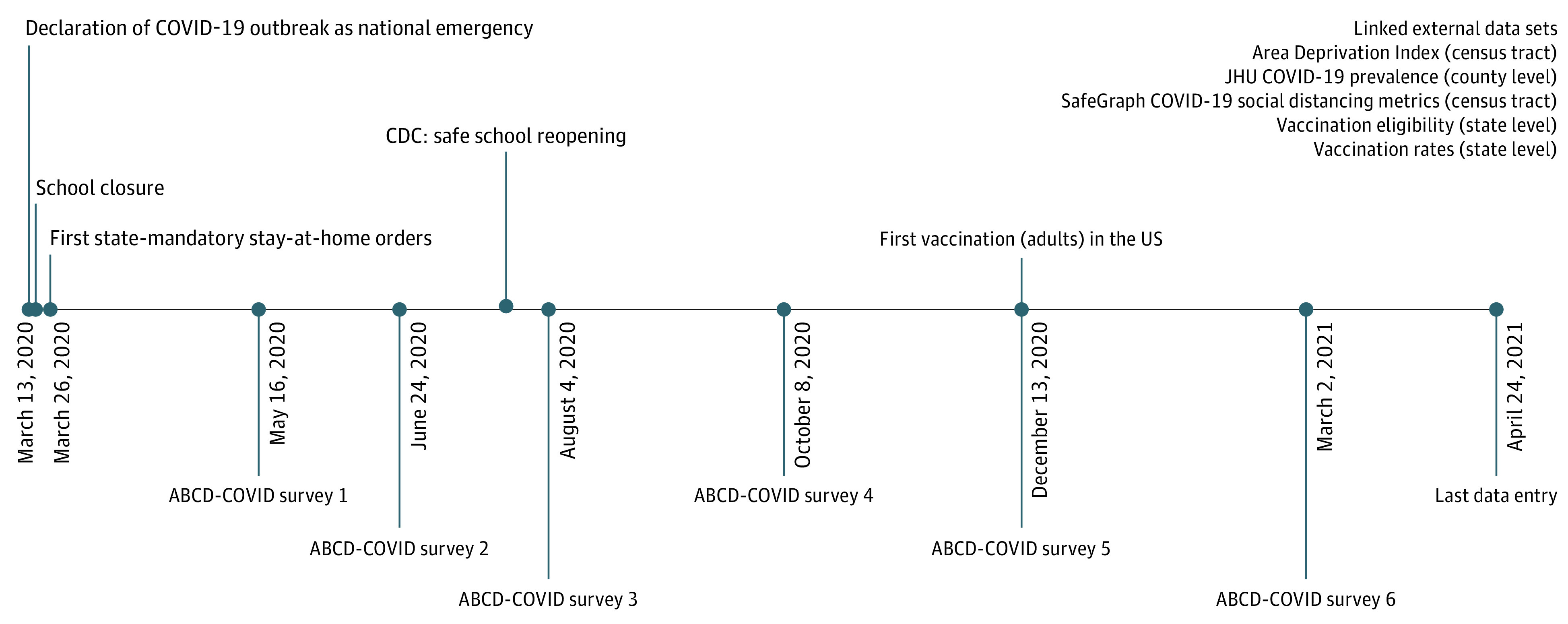
Study Timeline, Adolescent Brain Cognitive Development (ABCD) Study COVID-19 Survey, Linked Database, and US COVID-19 Responses The text labels below the timeline and in the right upper corner represent the ABCD COVID-19 surveys and assembled external data sets, respectively. The text above the timeline represents selected federal- and state-level responses. Adult vaccination rollout occurred on December 13, 2020. The ABCD COVID-19 Rapid Response Research consisted of online surveys disseminated on May 16 to May 22, 2020 (n = 7240, survey 1), June 24 to June 27, 2020 (n = 7554, survey 2), August 4 to August 5, 2020 (n = 6852, survey 3), October 8, 2020 (n = 6688, survey 4), December 13, 2020 (n = 6068, survey 5), and March 2, 2021 (n = 5929, survey 6). Because the surveys did not restrict expiration date, participants could complete the surveys on different dates other than the distribution dates. The last completed questionnaire was returned on April 24, 2021 (Figure 2; eMethods 1 and 2 in the Supplement). CDC indicates US Centers for Disease Control and Prevention; JHU, Johns Hopkins University.

Data for this cohort study were drawn from the Adolescent Brain Cognitive Development (ABCD) study.^33^ Baseline data were based on a stratified probability sample of schools from 21 testing sites in the US and 11 878 children recruited from 2016 to 2018. Previous studies^34,35^ have detailed ABCD study design. To incorporate individual- and structural-level SDoH, we assembled a longitudinal data set, comprising 6 main components (Figure 2; eMethods 1 and 2 in the Supplement): (1) ABCD COVID-19 Rapid Response Research on child mental health and experiences reported by children and parents/caregivers through 6 wave surveys between May 16, 2020, and April 24, 2021; (2) ABCD COVID-19 geocoded data on individual-level time-varying SDoH^36^; (3) ABCD child residential history data on structural-level preexisting SDoH; (4) ABCD baseline children’s sociodemographic characteristics (ABCD 4.0 release); (5) dates where all adults aged 18 years or older were eligible for vaccines, cross-referenced from *The New York Times*^37^ and *US News & World Report*^38^; and (6) the US Centers for Disease Control and Prevention COVID-19 Vaccine Tracker.^39^ After sample selection (eg, at least 3 repeated measures, eFigure 1 in the Supplement), the study sample contained 44 958 observations. We used all records with available information. The ABCD sample is not nationally representative. However, the ABCD cohort, when compiled with the COVID-19 and geocoded data, enables our examination in multilevel SDoH of mental health in the largest US child sample, tracking 47 weeks during COVID-19, which distinguishes our study from previous investigations. All measures are described in eTable 2 in the Supplement.

All parents/caregivers provided written informed consent and children provided verbal assent to research protocols approved by either a central or local institutional review board. We obtained a data-use certificate from the National Institute of Mental Health Data Archive use agreement. This study followed the Strengthening the Reporting of Observational Studies in Epidemiology (STROBE) reporting guidelines.

#### Child Mental Health

We included 4 mental health measures to capture psychopathology (stress, COVID-19–related worry, sadness) and well-being (positive affect) based on the dual-factor mental health model.^40^

Perceived stress was measured with the 4-item version of the Perceived Stress Scale (PSS).^41^ Children were asked for past-month frequency of stressful situations (eg, “How often have you felt that things were going your way?”) on a 5-point Likert scale (from 0 = never to 4 = very often). Two items (Q2, Q4) were reversely scored, and scores were summed on the PSS-4. COVID-19–related worry was measured by 1 question asking children, “How worried have you been about coronavirus (COVID-19)?” on a 5-point Likert scale (from 1 = not at all to 4 = extremely).

Sadness (8 items; eg, feeling lonely, sad, unhappy) and positive affect (9 items; eg, feeling happiness, joy, serenity, peace) were measured using the National Institutes of Health (NIH)–Toolbox emotion battery on a 5-point scale (from 1 = never to 5 = almost always). We followed the NIH-Toolbox Scoring and Interpretation Guide^42^ and calculated the uncorrected standard score (T score). Higher scores represented more stress, COVID-related worry, sadness, and positive affect.

#### Social Determinants of Health

We measured preexisting (long-standing features presented before COVID-19) and time-varying SDoH (pandemic-related changes in conditions) at individual and structural levels based on our theoretical framework (Figure 1).

Preexisting SDoH at the individual level included baseline parent-reported (1) annual household income (<$50 000, $50 000-$100 000, ≥$100 000); (2) highest level of parental education (<bachelor’s degree, ≥bachelor’s degree); and (3) parent marital status (married, widowed, divorced, separated, never married, cohabitated).

At the structural level, we used the census tract–level Area Deprivation Index (ADI), which was calculated from the 2011 to 2015 5-year American Community Survey estimates of children’s baseline residential address. The ADI is a composite-weighted metric of 17 neighborhood disadvantage indicators (eg, poverty, unemployment, family income, low education; eTable 1 in the Supplement). ADI was used because it does not include indirect measures of race and ethnicity, minimizing potential confounding. ADI national percentiles were aggregated into 3 levels: most, intermediate, and least deprived. The original proposed ADI was contributed by the code for computing and merging ADI (and its national percentile) with ABCD data are available at the ABCD Data Analytics and Informatics Resource Center.^43^

Time-varying SDoH at the individual level included parent-reported COVID-19–related (1) food insecurity (whether their families “needed food but couldn't afford to buy it or couldn't afford to get out to get it?” [cannot afford], and “worried about whether your food would run out before you could get more” [worry about food]), (2) unemployed parents/caregivers (whether there were family members lost wages and/or job owing to COVID-19), and (3) disrupted health care access (how COVID-19 changed their medical health care and mental health treatment access).

At the structural level, SDoH that potentially amplified disparities in child mental health included (1) county-level COVID-19 infection and mortality rates from Center of Systems Science and Engineering at Johns Hopkins University,^44^ (2) census block-level Social Distancing Metrics^45^ from SafeGraph (median distance traveled from home, time at home, devices detected completely at home, part-time and full-time work behaviors were used; all were proxies for the ability to social distance, as essential workers had to travel to work and experienced greater exposure),^46^ and (3) state-level timeline of all adults eligible for vaccination (before April 2021, April 1-14, after April 15, 2021) and rates of fully vaccinated adults (4 quantiles) to account for geographic heterogeneity (eMethods 3 in the Supplement).

#### Demographic Variables

Baseline parent-reported children’s demographic characteristics: age (10-11 years, 12-15 years), biological sex (male or female), race and ethnicity (Asian, Black, Hispanic, White, and other/multiracial). The 5-level race and ethnicity variable is consistent with the US Office of Management and Budget (OMB) Standards for presenting data on race and ethnicity.^34^

#### Time Variables

Temporal trends were anchored by interview days and survey numbers. Given nonlinear changes in mental distress observed previously and model fit assessment (eMethods 4 in the Supplement),^47,48,49^ interview dates were continuous variables representing the number of days from May 16, 2020, to April 24, 2021 (range, 0-343 days). Categorical survey numbers (1-6) were used for variations in survey launching periods.

### Statistical Analysis

Descriptive statistics for the COVID-19 survey sample were compared with the ABCD main sample. Multilevel generalized linear mixed models (GLMM) were used to examine SDoH on child mental health trajectories (eMethods 4 in the Supplement). A multilevel framework is more flexible when incorporating time variation in follow-up waves,^35,50,51^ as applied in previous similarly designed studies.^52^ GLMM was fitted to estimate fixed and random effects of intercept (baseline mental health) and slope (change per assessment) by days of follow-up, allowing an unstructured covariance matrix of intercept and slope. Random effects included random intercepts for study site and participant identification, adjusting for the nested nature of ABCD. We did not use the American Community Survey poststratification propensity weights because the purpose of our study was not to obtain population-representative estimates but to examine site-specific SDoH across 17 states (eMethods 5 in the Supplement). Race and ethnicity SDoH interactions were tested because there are racial and ethnic differences in the associations between SDoH and mental health.^53^

We modeled mental health trajectories using restricted cubic splines with 95% CIs relative to the median. Five knots (5, 27.5, 50, 72.5, 95 percentiles) were created using the Harrell method (eMethods 4 in the Supplement).^54^ Missing data were uncommon. Categorical factors were effects-coded, and continuous factors were centered.^55^ We conducted sensitivity analyses using alternative samples, measures, models (eMethods 6 in the Supplement).^56^ Statistical significance was determined by a 2-sided *P* value <.05. Analyses and visualization were conducted using Stata, version 17 (StataCorp), R (R Foundation for Statistical Computing), and Python, version 3.9 (Python Software Foundation).

## Results

### Descriptive Characteristics of the Child Participants

At baseline, the longitudinal sample included 8493 children (mean [SD] age, 9.93 [0.63] years; 5011 girls [47.89%]; 3482 boys [41.00%]; 245 Asian [2.34%], 1213 Black [11.59%], 2029 Hispanic [19.39%], 5851 White [55.93%], and 1124 children of other/multiracial ethnicity [10.74%]). A total of 4544 children (47.23%) lived in lower-income families (<$50 000 per year), and 1709 children (31.15%) lived in the most economically deprived areas (Table). Black children disproportionately lived in the most economically deprived areas (eFigure 2 in the Supplement). Compared with the overall ABCD sample, the COVID-19 Rapid Response Research sample contained more White children, children with married parents, children with parents/caregivers with a bachelor’s degree, and children living in higher-income families (>$100 000 per year) and less-deprived areas (ie, bottom 30% of the ADI, representing less poverty and better educated residents). There were decreases in children worried about food (from 1714 [17.53%] to 901 [13.15%]) and families who cannot afford food (from 600 [6.15%] to 320 [4.68%]), but persistently high rates of COVID-19 pandemic–related unemployed parents/caregivers and disrupted health care service access. Social distancing decreased from May 2020 to August 2020 but subsequently increased until the end of 2020 before a second decrease. Approximately 43% (3158 of 7320) of children lived in states where all adults were eligible for vaccination from April 1 to April 14, 2021, and 30% (2411 of 7320) lived in states ranked in the second quantile of fully vaccinated adult rates.

**Table.** Demographic Characteristics, Social Determinants of Health (SDoH), and Mental Health of the Study Sample From the Adolescent Brain Cognitive Development (ABCD) Study, May 2020 to April 2021^a^

Characteristic	Survey 1 (May 16-22, 2020)	Survey 2 (Jun 24-27, 2020)	Survey 3 (Aug 4-5, 2020)	Survey 4 (Oct 8, 2020)	Survey 5 (Dec 13, 2020)	Survey 6 (Mar 2, 2021)	ABCD main sample at baseline
Demographic characteristics							
Age, mean (SD), y	9.93 (0.63)	9.92 (0.62)	9.91 (0.62)	9.92 (0.63)	9.93 (0.63)	9.91 (0.62)	9.91 (0.61)
Age group, No. (%)							
8-9 y	5797 (55.40)	5621 (55.71)	4767 (56.33)	4384 (55.70)	3876 (55.28)	4659 (56.13)	6678 (56.38)
10-11 y	4667 (44.60)	4468 (44.29)	3695 (43.67)	3487 (44.30)	3135 (44.72)	3641 (43.87)	5166 (43.62)
Sex, No. (%)							
Male	5453 (52.11)	5393 (53.45)	4384 (51.81)	4003 (50.86)	3679 (52.47)	4229 (50.95)	6182 (52.20)
Female	5011 (47.89)	4696 (46.55)	4078 (48.19)	3868 (49.14)	3332 (47.53)	4071 (49.05)	5662 (47.80)
Race and ethnicity, No. (%)							
Asian	245 (2.34)	205 (2.03)	203 (2.40)	172 (2.19)	177 (2.53)	216 (2.60)	250 (2.11)
Black	1213 (11.59)	1235 (12.24)	978 (11.56)	875 (11.12)	741 (10.57)	845 (10.18)	1778 (15.01)
Hispanic	2029 (19.39)	1809 (17.94)	1538 (18.19)	1460 (18.56)	1324 (18.89)	1576 (18.99)	2406 (20.32)
Other/multiracial^b^	1124 (10.74)	1127 (11.17)	884 (10.45)	817 (10.38)	700 (9.99)	837 (10.09)	1243 (10.50)
White	5851 (55.93)	5710 (56.61)	4854 (57.40)	4544 (57.75)	4067 (58.03)	4824 (58.13)	6165 (52.06)
Preexisting SDoH (individual), No. (%)							
Household income, $							
<50 000	2332 (24.24)	2423 (25.67)	1836 (23.39)	1778 (24.21)	1566 (24.01)	1799 (23.25)	3215 (29.69)
≥50 000	2746 (28.54)	2840 (30.09)	2322 (29.59)	2212 (30.12)	1907 (29.24)	2300 (29.72)	3066 (28.32)
>100 000	4544 (47.23)	4175 (44.24)	3690 (47.02)	3353 (45.66)	3048 (46.74)	3640 (47.03)	4547 (41.99)
Parent education							
≥Bachelor’s degree	6813 (65.23)	6580 (65.27)	5686 (67.28)	5191 (66.01)	4689 (66.96)	5540 (66.80)	7035 (59.47)
<Bachelor’s degree	3632 (34.77)	3501 (34.73)	2765 (32.72)	2673 (33.99)	2314 (33.04)	2754 (33.20)	4795 (40.53)
Parent marital status							
Married	7576 (72.85)	7113 (70.90)	6209 (73.74)	5676 (72.31)	5161 (73.92)	6106 (73.96)	7968 (67.82)
Widowed	51 (0.49)	73 (0.73)	80 (0.95)	63 (0.80)	42 (0.60)	60 (0.73)	97 (0.83)
Divorced	925 (8.90)	903 (9.00)	705 (8.37)	718 (9.15)	634 (9.08)	750 (9.08)	1078 (9.18)
Separated	330 (3.17)	371 (3.70)	259 (3.08)	241 (3.07)	215 (3.08)	247 (2.99)	464 (3.95)
Never married	988 (9.50)	1065 (10.61)	758 (9.00)	769 (9.80)	628 (8.99)	688 (8.33)	1453 (12.37)
Living with partner	529 (5.09)	508 (5.06)	409 (4.86)	383 (4.88)	302 (4.33)	405 (4.91)	688 (5.86)
Preexisting SDoH (structural), No. (%)							
Area Deprivation Index^c^							
Least deprived	1962 (35.76)	1782 (33.45)	1573 (34.88)	1423 (33.91)	1329 (34.85)	1607 (35.64)	2940 (35.56)
Intermediate deprived	1815 (33.08)	1791 (33.62)	1583 (35.10)	1431 (34.10)	1278 (33.51)	1523 (33.78)	2768 (33.48)
Most deprived	1709 (31.15)	1754 (32.93)	1354 (30.02)	1343 (32.00)	1207 (31.65)	1379 (30.58)	2559 (30.95)
Time-varying SDoH (individual), No. (%)							
Food insecurity							
Worried about food	1714 (17.53)	1378 (15.12)	1148 (14.43)	955 (14.35)	824 (13.70)	901 (13.15)	NA
Cannot afford food	600 (6.15)	543 (5.95)	482 (6.07)	375 (5.64)	320 (5.34)	320 (4.68)	NA
Unemployed family member	4594 (47.25)	4382 (48.30)	3899 (49.23)	3148 (47.44)	2828 (47.24)	3137 (46.04)	NA
Disrupted health service access^d^							
Medical health care	3786 (75.96)	4697 (76.03)	3795 (76.64)	3652 (76.53)	3386 (77.09)	3288 (76.86)	NA
Mental health treatment	1672 (34.20)	2174 (35.33)	1693 (34.20)	1622 (33.99)	1486 (33.83)	1424 (33.29)	NA
Time-varying SDoH (structural), No. (%)							
COVID-19 prevalence^e^							
Cases	1269.37 (1733.60)	1418.94 (1746.49)	1655.07 (1839.90)	1813.21 (1902.86)	2156.35 (2246.08)	1843.78 (2063.35)	NA
New cases	15.67 (20.62)	17.38 (21.08)	18.79 (21.65)	19.02 (22.07)	24.22 (28.30)	20.32 (24.80)	NA
Deaths	33.48 (38.76)	37.45 (41.28)	39.87 (41.48)	42.45 (42.05)	44.51 (44.51)	41.87 (42.61)	NA
New deaths	0.28 (0.49)	0.23 (0.38)	0.27 (0.53)	0.25 (0.60)	0.27 (0.51)	0.26 (0.48)	NA
Social distance^f^							
Distance traveled	2514.79 (8060.64)	2416.21 (3833.93)	2440.65 (6932.65)	2512.49 (6318.27)	2525.63 (10504.66)	2515.02 (9619.17)	NA
Home dwelling	806.60 (271.63)	762.78 (266.08)	764.56 (265.36)	762.69 (259.49)	784.47 (276.62)	779.07 (266.86)	NA
Device at home	0.34 (0.10)	0.33 (0.10)	0.33 (0.10)	0.32 (0.10)	0.34 (0.10)	0.33 (0.10)	NA
Full-time job	0.04 (0.03)	0.04 (0.03)	0.04 (0.03)	0.04 (0.03)	0.04 (0.03)	0.04 (0.03)	NA
Part-time job	0.06 (0.04)	0.07 (0.04)	0.07 (0.04)	0.07 (0.04)	0.07 (0.04)	0.07 (0.04)	NA
Vaccination^g^							
Adults eligible for vaccination							
Before April 2021	1751 (23.92)	1892 (24.96)	1688 (24.62)	1638 (24.50)	1464 (24.11)	1430 (24.10)	NA
April 1-14, 2021	3158 (43.14)	3314 (43.72)	2974 (43.38)	2950 (44.12)	2653 (43.70)	2564 (43.21)	NA
After April 15, 2021	2411 (32.94)	2374 (31.32)	2194 (32.00)	2099 (31.39)	1954 (32.19)	1940 (32.69)	NA
Adults fully vaccinated, quantile							
First	1817 (24.82)	2007 (26.48)	1789 (26.09)	1725 (25.80)	1512 (24.91)	1493 (25.16)	NA
Second	2146 (29.32)	2057 (27.14)	1845 (26.91)	1774 (26.53)	1647 (27.13)	1607 (27.08)	NA
Third	1473 (20.12)	1596 (21.06)	1460 (21.30)	1440 (21.53)	1333 (21.96)	1260 (21.23)	NA
Fourth	1884 (25.74)	1920 (25.33)	1762 (25.70)	1748 (26.14)	1579 (26.01)	1574 (26.53)	NA
Mental health							
Perceived stress	5.57 (2.97)	5.28 (2.82)	5.08 (2.81)	5.60 (2.96)	5.73 (2.94)	5.71 (3.03)	NA
COVID-19 worry	2.39 (1.05)	2.36 (1.02)	2.29 (0.99)	2.33 (1.01)	2.34 (1.05)	2.21 (1.01)	NA
NIH-Toolbox sadness T scores^h^	49.34 (10.99)		48.30 (11.11)		49.62 (12.49)		
NIH-Toolbox positive affect T scores^h^		45.59 (13.30)		45.26 (13.30)		45.33 (12.97)	NA

Abbreviations: NA, not applicable; NIH, National Institutes of Health.

^a^ Details of each variable measure can be found in eTable 2 in the Supplement.

^b^ The other/multiracial category indicates that no specific racial and ethnic group was identified, and primary caregivers were allowed to choose multiple racial subgroups for children.

^c^ Area Deprivation Index was used to quantify neighborhood disadvantage and specifically test its association with racial disparities in COVID-19 positivity (detailed descriptions in eTable 1 in the Supplement).

^d^ Health service access includes 2 categories: no change and disrupted access, including mild (appointments moved to telehealth), moderate (delays or cancellations in appointments and/or delays in getting prescriptions; changes have minimal impact on health), and severe (unable to access needed care).

^e^ County-level, normalized by population, per 100 000. Data were geocoded using COVID-19 infection and mortality rates from the Center of Systems Science and Engineering at Johns Hopkins University.

^f^ Distance traveled, home dwelling, and device at home were measured by census block/meters; full-time job and part-time job social mobilities were measured by census block ratio. Social distancing data were from Social Distancing Metrics (SafeGraph).

^g^ Vaccination eligibility is cross-sourced from *The New York Times* and *US News & World Report* based on information from state and county health departments.

^h^ Sadness and positive affect were measured using 8 items (sadness) and 9 items (positive affect) from the NIH Toolbox emotion battery. Each item was administered a 5-point scale (from 1 = never to 5 = almost always). We followed the NIH Toolbox Scoring and Interpretation Guide^29^ and calculated the uncorrected standard score (T score). Higher scores indicate more sadness (eg, feeling lonely, sad, unhappy) and more positive affect (eg, feeling happiness, joy, serenity, peace).

### Vaccination and Trends in Child Mental Health Trajectories

The mean (SD) of perceived stress and sadness scores declined from survey 1 during May 16, 2020 (5.57 [2.97]), to May 22, 2020 (49.34 [10.99]), to survey 3 during August 4, 2020 (5.08 [2.81]), to August 5, 2020 (48.30 [11.11]). The mean of COVID-19–related worry and positive affect decreased steadily over time (Table).

**Figure 3.**
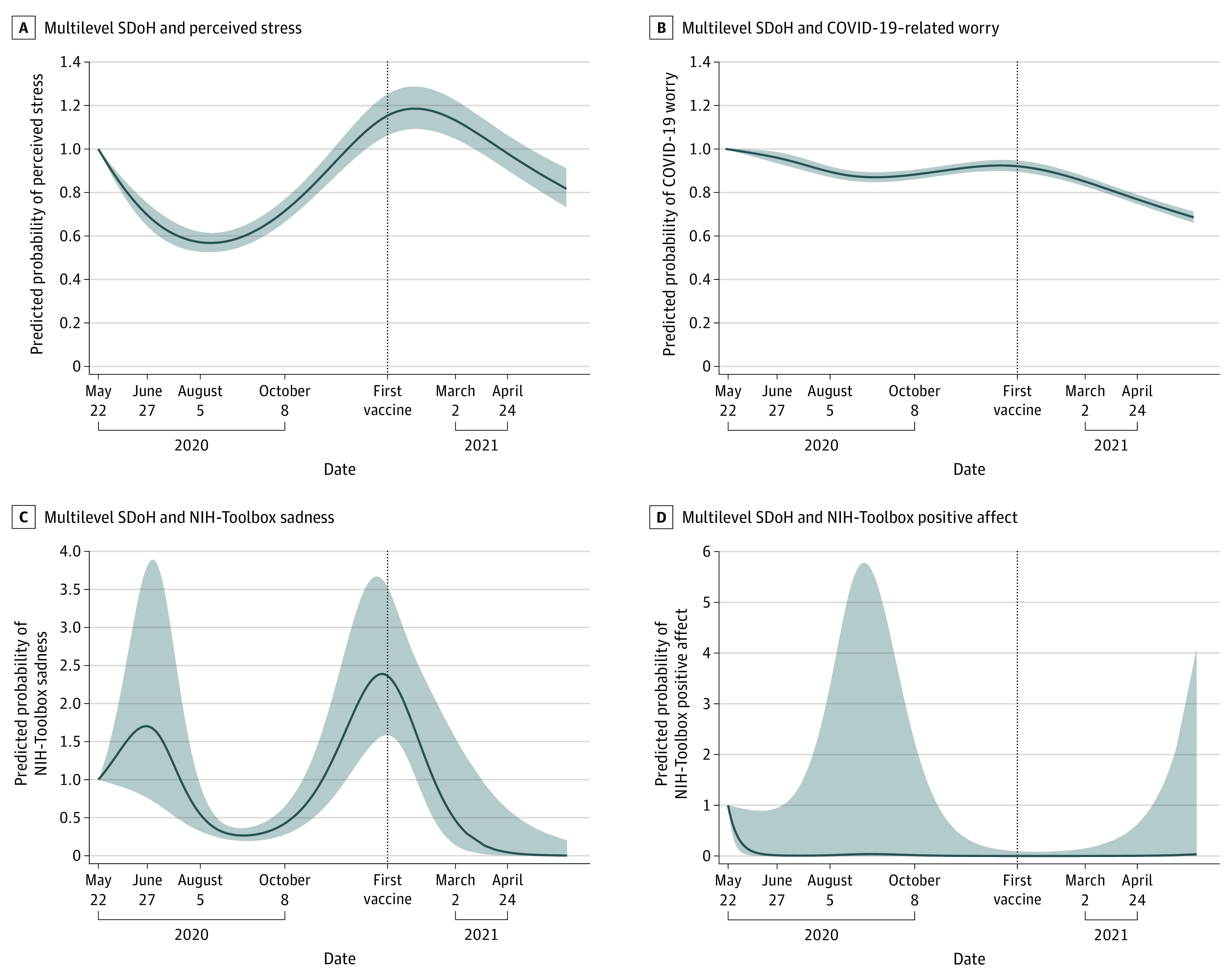
Predicted Probabilities of Self-reported Perceived Stress, COVID-19 Worry, National Institutes of Health (NIH)–Toolbox Sadness, and NIH-Toolbox Positive Affect with 95% CIs by Date of Survey Completion Among US Children, Based on Restricted Cubic Spline Regression Results from 4 separate restricted cubic spline regression. A, Results for multilevel social determinants of health (SDoH) and perceived stress. B, Results for multilevel SDoH and COVID-19–related worry. C, Results for multilevel SDoH and NIH-Toolbox sadness. D, Results for multilevel SDoH and NIH-Toolbox positive affect. Shaded areas denote 95% CIs. The gray-shaded line notates the date of adult vaccination rollout.

Estimated values from restricted cubic spline regressions revealed distinct mental health trajectories (Figure 3). Predicted perceived stress and COVID-19–related worry declined from May to August 5, 2020, increased in the fall of 2020, but decreased continuously after adult vaccination started (December 13, 2020). Children’s sadness increased in the first survey (May 22-June 27, 2020), decreased during the summer of 2020, rose in the fall to a peak around December 2020, and then decreased.

### Preexisting and Time-Varying SDoH Associations With Child Mental Health Trajectories

**Figure 4.**
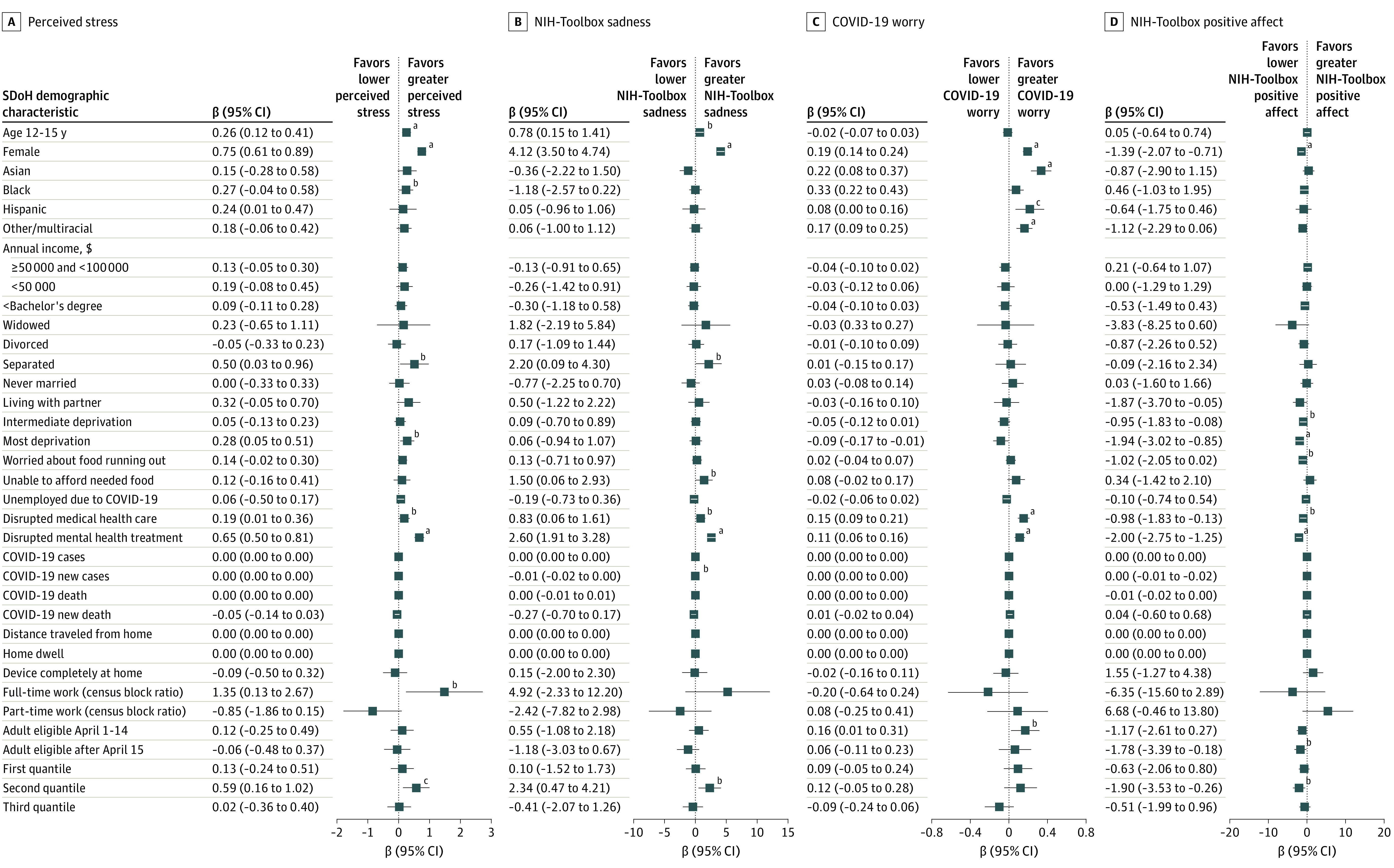
Results From Multilevel Generalized Mixed-Effect Modeling Results from 4 separate multilevel generalized mixed-effect modeling. A, Results for multilevel social determinants of health (SDoH) and perceived stress. B, Results for multilevel SDoH and National Institutes of Health (NIH)–Toolbox sadness. C, Results for multilevel SDoH and COVID-19 pandemic–related worry. D, Results for multilevel SDoH and NIH-Toolbox positive affect. Error bars indicate 95% CIs. The other/multiracial subcategory was defined when primary caregivers did not identify a specific racial and ethnic group, and primary caregivers were allowed to choose multiple racial subgroups for children. ^a^*P* < .001. ^b^*P* < .05. ^c^*P* < .01.

An adjusted GLMM (Figure 4 and eTable 3 in the Supplement) found that older children aged 12 to 15 years (β = 0.26; 95% CI, 0.12-0.41; *P* < .001), girls (β = 0.75; 95% CI, 0.61-0.89; *P* < .001), Hispanic children (β = 0.24; 95% CI, 0.01-0.47; *P* = .04), children living with separated parents (β = 0.50; 95% CI, 0.03-0.96; *P* = .04), and children living in the most economically deprived neighborhoods (β = 0.28; 95% CI, 0.05-0.51; *P* = .02) reported increasing and greater stress than children who were younger, boys, White, or living with married parents. Older children (β = 0.78; 95% CI, 0.15-1.41; *P* = .02), girls (β = 4.12; 95% CI, 3.50-4.74; *P* < .001), and children living with separated parents (β = 2.20; 95% CI, 0.09-4.30; *P* = .04) had more NIH-Toolbox sadness. Compared with White children, COVID-19 pandemic–related worry was higher among Asian children (β = 0.22; 95% CI, 0.08-0.37; *P* = .003), Black children (β = 0.33; 95% CI, 0.22-0.43; *P* < .001), children of other/multiracial ethnicity (β = 0.17; 95% CI, 0.09-0.25; *P* < .001), and children with disrupted medical health care (β = 0.15; 95% CI, 0.09-0.21) and disrupted mental health treatment (β = 0.11; 95% CI, 0.06-0.16).

Higher stress scores were associated with disrupted access to medical health care (β = 0.19; 95% CI, 0.01-0.36; *P* = .04) and to mental health treatment (β = 0.65; 95% CI, 0.50-0.81; *P* < .001), and with living in communities with more full-time working adults (β = 1.35; 95% CI, 0.13-2.67; *P* = .04). Children living with families unable to afford needed food (β = 1.50; 95% CI, 0.06-2.93; *P* = .04) experienced higher and increasing trajectories of sadness.

Living in states with lower fully vaccinated rates was associated with increasing stress (β = 0.59; 95% CI, 0.16-1.02; *P* = .007), increasing NIH-Toolbox sadness (β = 2.34; 95% CI, 0.47-4.21; *P* = .01), and decreasing NIH-Toolbox positive affect (β = −1.90; 95% CI, −3.53 to −0.26; *P* = .02). Living in states with later dates for all adults being eligible for vaccination was associated with increased COVID-19–related worry (β = 0.16; 95% CI, 0.01-0.31; *P* = .03) and decreased positive affect (β = −1.78; 95% CI, −3.39 to −0.18; *P* = .03).

### Association of SDoH With Asian, Black, and Hispanic Race and Ethnicity

Restricted cubic spline regressions stratified by race, ethnicity, and SDoH indicated steeper increases in perceived stress trajectories in older Hispanic children, children living in deprived areas, experiencing food insecurity, and households with unemployed parent/caregivers (eFigures 3-5 in the Supplement).

We observed race-SDoH interaction associations for 4 mental health outcomes (eFigures 6-25 in the Supplement): steeper increases in COVID-19–related worry in Black and Hispanic children and children of other/multiracial ethnicity whose parents had less than bachelor’s degrees (eFigure 7 in the Supplement), Black and Hispanic children living in lower-income households (<$50 000 per year, eFigure 6 in the Supplement), and Black children living in the most deprived neighborhoods (eFigure 9 in the Supplement). Asian children whose families could not afford food had a steeper increase in perceived stress and sadness than White children (eFigure 11 in the Supplement). Hispanic children reported decreased sadness (β = −3.20; 95% CI, −5.93 to −0.47; *P* = .02) and increased positive affect (β = 4.03; 95% CI, 1.01-7.04; *P* = .009) when living in states where adults became eligible for vaccines earlier (eFigure 24 in the Supplement). Black children reported a steeper increase in COVID-19–related worry if they were from states lagging in vaccinations (eFigure 25 in the Supplement). Our findings proved robust in sensitivity analyses (eMethods 6 in the Supplement).

## Discussion

In this cohort study, we found that children experienced decreasing-increasing trajectories of stress and sadness from May 16 to December 12, 2020, followed by a consistent decrease after December 13, 2020, which coincided with vaccination rollout and new government policies for health and the economy beginning in January 2021. Preexisting (living in deprived areas) and time-varying (food insecurity, unemployed family members, disrupted health care, living in neighborhoods with more adults working full-time away from home, vaccinations) SDoH and race-SDoH interactions were associated with child mental health disparities.

### Individual-Level SDoH

Trajectories of stress and COVID-19–related worry were associated with time-varying SDoH. Previous studies reported greater depression in adults experiencing food insecurity.^57,58,59^ We found that children in households experiencing food insecurity also had increasing sadness and decreasing positive affect.^60^ Public health policies should consider improving nutritional assistance programs, such as the Supplemental Nutrition Assistance Program and Women, Infants, and Children, expanding the amount and duration of unemployment insurance and housing subsidies, extending eviction moratoriums,^4^ and increasing universal minimum-wage to reduce food insecurity for improving child mental health.^60,61^

Children who experienced disrupted medical health care and mental health treatment reported worsening mental health outcomes. School closures, lack of access to telehealth, and disrupted community-based pediatric care may contribute to adverse child mental health.^23^ Expanding health care insurance coverage, minimizing cost-sharing (out-of-pocket expense), supporting community behavioral health facilities, improving value-based care, and developing broadband infrastructure to expand telehealth may reduce inequalities.^62^

### Geographic Heterogeneity and Structural SDoH

Children living in areas with more adults working full-time, a possible proxy for a higher concentration of essential workers and inability to work from home,^63^ reported greater stress. Low-wage essential workers had fewer employment protections (eg, fewer paid sick leave, lack of unemployment insurance, risk of COVID-19 exposures while on the job).^64^ This additional stress may have affected their children.^52^ Neighborhoods with high full-time working rates may have been low-income areas with greater racial and ethnic disparities in COVID-19 infections/mortality, barriers to social distancing, and vaccination uptake owing to lack of trust in health directives and structural racism.^65^ Public policies should address the needs of under-resourced communities, minoritized households, and parents who were essential workers. Basic needs, such as paid sick leave, expanded insurance coverage, employment protection, financial assistance, Earned Income Tax Credit, and rent stabilization, should be considered along with childcare resources and direct food assistance.^4,65^

This is the first study, to our knowledge, that found a possible association between children’s mental health and adult COVID-19 vaccination.^66^ Less anxiety and depression were observed in adults in US states with higher COVID-19 vaccination rates.^67^ This suggests the potential benefit of vaccinations extends to child mental health.^30^ States with more vaccinated adults may indicate better health literacy, trust in vaccines, and earlier and equitable access to vaccination.^68^ Vaccination acts as a pandemic-related structural SDoH for child mental health by potentially reducing uncertainty, fear, and stress, while improving health outcomes in vaccinated people.^21^

### Racial and Ethnic Differences and Association With SDoH

Within the syndemics of COVID-19, SDoH, and structural racism, there are intersectional risks on child mental health trajectories by race and ethnicity. Black and Hispanic children reported greater stress, sadness, and COVID-19–related worry when living in economically deprived areas and having families with food insecurity and unemployed parents (Figure 4 and eTable 3 in the Supplement). Differences in risk and resilience profiles owing to racism and structural inequities demand health and economic policy adjustments.^69,70^ Discrimination, racism, and bias may magnify the association of SDoH with child mental health disparities as indicated in previous ABCD studies in baseline^71^ and during the COVID-19 pandemic.^72^ After a call from the US Surgeon General,^73^ it is imperative to systematically quantify, uncover, and deconstruct the multilevel and intersectional mechanisms of disparities to inform nuanced solutions to mental health disparities in children.

### Research and Policy Implications to Address Child Mental Health Disparities

This study highlighted the importance of long-term investigation of the trajectories of children’s mental health given the ongoing pandemic, multilevel SDoH, and stressors/trauma that may be associated with child mental health. Older children; girls; Asian, Black, and Hispanic children; children of other/multiracial ethnicity; children who experienced food insecurity and disrupted health care access; and children living in economically deprived areas, areas with high full-time work behaviors, and low vaccination rates have disproportionate outcomes associated with the COVID-19 pandemic. Future research should address key time-varying (eg, food insecurity, full-time/part-time working conditions) and mechanistic pathways linking SDoH and children’s mental health trajectories when future waves of data become available. There is a need for studies in children and parents to understand factors associated with perceived stress, sadness, and worry.

Clinicians, psychiatrists, and social work practitioners should consider the SDoH of child mental health during the COVID-19 pandemic and partner with community agencies to address the structural barriers. Integrating structural competency and clinical practice may facilitate institutional and policy interventions to bridge the gap between SDoH and clinical strategies.^74^ Multipronged, upstream, and equity-responsive approaches are needed to reduce structural barriers to food, mental health services, housing, and employment benefits.^57^

### Strengths and Limitations

Study strengths included the use of large, population-based, longitudinal data measuring child mental health outcomes repeatedly over 47 weeks during the COVID-19 pandemic. The study extended previous knowledge about children’s mental health under COVID-19 by linking individual-level and structural-level SDoH and incorporating time-varying factors that capture pandemic-related dynamics.

This study also had limitations. ABCD measures are self-reported, subject to social desirability and recall biases. Biases were mitigated using multiple mental health measures (eg, PSS, NIH Toolbox). Second, there are multiple interaction effects within SDoH and between SDoH, race, ethnicity, sex, and other health disparity status.^72^ Other psychological disorders and brain development factors are potentially important. We could not include all possible factors as our main purpose was to evaluate the SDoH relationship with child mental health disparities. Third, data on child mental health between the appearance of COVID-19 in the US (December 2019) and the start of school closure, were not available. Future studies are needed to reproduce time-varying SDoH influences in larger populations to be generalized to the entire US child population.

## Conclusions

Results of this cohort study suggest a disproportionately adverse association of the COVID-19 pandemic with child mental health among racial and ethnic minority groups, which may be improved by addressing modifiable individual (food insecurity, unemployment, health services, parental supervision) and structural (area deprivation, job protection, vaccination) SDoH.

Identifying and addressing preexisting and time-varying social determinants of mental health in children, in tandem with empowering the most marginalized populations and communities, appear critical for addressing mental health disparities. Children from families facing food insecurity, disrupted health care access, living in areas with greater full-time working behaviors, and lower vaccination rates, experienced adverse mental health outcomes. Public policies targeting food assistance, unemployment insurance, worksite parental leave policies, and health care access may lead to more sustainable and equitable paths to reduce mental health disparities. Child mental health was associated with an improvement at the time of adult vaccine rollout. Enhancing vaccination uptake through place-based allocations, targeted community outreach, and investment to reduce structural barriers and build trust, may potentially improve child mental health.

## Notes

Supplement. **eMethods 1.** ABCD Study Design and ABCD COVID Rapid Response Research (RRR) Survey **eMethods 2.** Additional Data Sources **eMethods 3.** Consideration of Geographic Heterogeneity in Vaccine **eMethods 4.** Generalized Linear Mixed Models (GLMM) and Restricted Cubic Splines **eMethods 5.** Consideration of Clustering and Population-Based Analysis of the Adolescent Brain Cognitive Development (ABCD) Study Data **eMethods 6.** Sensitivity Analysis **eFigure 1.** Flowchart of Study Sample Inclusion and Exclusion **eFigure 2.** Distribution of Race and Ethnicity by Area Deprivation Index (ADI) Deciles, 2020 in Study Sample **eFigure 3.** Predicted Probabilities of Self-Reported Perceived Stress With 95% CI by Date of Survey Completion among US Children, Based on Restricted Cubic Spline Regression, Stratified by Different Sociodemographic Characteristics **eFigure 4.** Predicted Probabilities of Self-Reported Perceived Stress With 95% CI by Date of Survey Completion Among US Children, Based on Restricted Cubic Spline Regression, Stratified by Individual-Level Time-Varying Social Determinants of Health (SDoH) Characteristics **eFigure 5.** Predicted Probabilities of Self-Reported Perceived Stress With 95% CI by Date of Survey Completion Among US Children, Based on Restricted Cubic Spline Regression, Stratified by Community-Level Preexisting and Time-Varying Social Determinants of Health (SDoH) Characteristics **eFigure 6.** Interaction Effects of Race/Ethnicity and Preexisting Individual SDoH (Household Income) on Child Mental Health^a^ **eFigure 7.** Interaction Effects of Race/Ethnicity and Preexisting Individual SDoH (Parent Education) on Child Mental Health^a^ **eFigure 8.** Interaction Effects of Race/Ethnicity and Preexisting Individual SDoH (Parent Marital Status) on Child Mental Health^a^ **eFigure 9.** Interaction Effects of Race/Ethnicity and Preexisting Structural SDoH (Area Deprivation Index) on Child Mental Health^a^ **eFigure 10.** Interaction Effects of Race/Ethnicity and Time-Varying Individual SDoH (Worried About Food) on Child Mental Health^a^ **eFigure 11.** Interaction Effects of Race/Ethnicity and Time-Varying Individual SDoH (Cannot Afford Food) on Child Mental Health^a^ **eFigure 12.** Interaction Effects of Race/Ethnicity and Time-Varying Individual SDoH (Unemployment Due to COVID-19 Pandemic on Economy) on Child Mental Health^a^ **eFigure 13.** Interaction Effects of Race/Ethnicity and Time-Varying Individual SDoH (Disrupted Medical Healthcare Access) on Child Mental Health^a^ **eFigure 14.** Interaction Effects of Race/Ethnicity and Time-Varying Individual SDoH (Disrupted Mental Health Treatment Access) on Child Mental Health^a^ **eFigure 15.** Interaction Effects of Race/Ethnicity and Time-Varying Structural SDoH (COVID-19 Case) on Child Mental Health^a^ **eFigure 16.** Interaction Effects of Race/Ethnicity and Time-Varying Structural SDoH (COVID-19 New Case) on Child Mental Health^a^ **eFigure 17.** Interaction Effects of Race/Ethnicity and Time-Varying Structural SDoH (COVID-19 Death) on Child Mental Health^a^ **eFigure 18.** Interaction Effects of Race/Ethnicity and Time-Varying Structural SDoH (COVID-19 New Death) on Child Mental Health^a^ **eFigure 19.** Interaction Effects of Race/Ethnicity and Time-Varying Structural SDoH (Distance Traveled from Home) on Child Mental Health^a^ **eFigure 20.** Interaction Effects of Race/Ethnicity and Time-Varying Structural SDoH (Home Dwelling) on Child Mental Health^a^ **eFigure 21.** Interaction Effects of Race/Ethnicity and Time-Varying Structural SDoH (Device Completely at Home) on Child Mental Health^a^ **eFigure 22.** Interaction Effects of Race/Ethnicity and Time-Varying Structural SDoH (Full-Time Work) on Child Mental Health^a^ **eFigure 23.** Interaction Effects of Race/Ethnicity and Time-Varying Structural SDoH (Part-Time Work) on Child Mental Health^a^ **eFigure 24.** Interaction Effects of Race/Ethnicity and Time-Varying Structural SDoH (Vaccination Eligibility) on Child Mental Health^a^ **eFigure 25.** Interaction Effects of Race/Ethnicity and Time-Varying Structural SDoH (Vaccination Rates) on Child Mental Health^a^ **eTable 1.** Area Deprivation Index (ADI) Item and Components **eTable 2.** Measures, Variables, and Recoding Mechanisms **eTable 3.** Results of Multilevel Generalized Mixed Models on Associations between Preexisting and Time-Varying Social Determinants of Health (SDoH) Factors^a^
eReferences

## References

[1] Abbasi J. Prioritizing physician mental health as COVID-19 marches on. JAMA. 2020;323(22):2235-2236. 32432665 10.1001/jama.2020.5205

[2] Berkowitz SA, Basu S. Unemployment insurance, health-related social needs, health care access, and mental health during the COVID-19 pandemic. JAMA Intern Med. 2021;181(5):699-702. 33252615 10.1001/jamainternmed.2020.7048 PMC8094006

[3] Amsalem D, Dixon LB, Neria Y. The coronavirus disease 2019 (COVID-19) outbreak and mental health: current risks and recommended actions. JAMA Psychiatry. 2021;78(1):9-10. 32579160 10.1001/jamapsychiatry.2020.1730

[4] Leifheit KM, Pollack CE, Raifman J, . Variation in state-level eviction moratorium protections and mental health among US adults during the COVID-19 pandemic. JAMA Netw Open. 2021;4(12):e2139585. 34919134 10.1001/jamanetworkopen.2021.39585 PMC8683968

[5] The World Health Organization. A conceptual framework for action on the social determinants of health. Accessed February 25, 2022. https://www.who.int/sdhconference/resources/ConceptualframeworkforactiononSDH_eng.pdf

[6] Newlove-Delgado T, McManus S, Sadler K, ; Mental Health of Children and Young People group. Child mental health in England before and during the COVID-19 lockdown. Lancet Psychiatry. 2021;8(5):353-354. 33444548 10.1016/S2215-0366(20)30570-8 PMC8824303

[7] Pierce M, Hope H, Ford T, . Mental health before and during the COVID-19 pandemic: a longitudinal probability sample survey of the UK population. Lancet Psychiatry. 2020;7(10):883-892. 32707037 10.1016/S2215-0366(20)30308-4 PMC7373389

[8] Panchal U, Salazar de Pablo G, Franco M, . The impact of COVID-19 lockdown on child and adolescent mental health: systematic review. Eur Child Adolesc Psychiatry. 2021;1-27. 34406494 10.1007/s00787-021-01856-w PMC8371430

[9] Li W, Wang Z, Wang G, . Socioeconomic inequality in child mental health during the COVID-19 pandemic: first evidence from China. J Affect Disord. 2021;287:8-14. 33761325 10.1016/j.jad.2021.03.009 PMC9754677

[10] Hawrilenko M, Kroshus E, Tandon P, Christakis D. The association between school closures and child mental health during COVID-19. JAMA Netw Open. 2021;4(9):e2124092. 34477850 10.1001/jamanetworkopen.2021.24092 PMC8417763

[11] Christakis DA, Van Cleve W, Zimmerman FJ. Estimation of US children’s educational attainment and years of life lost associated with primary school closures during the coronavirus disease 2019 pandemic. JAMA Netw Open. 2020;3(11):e2028786. 33180132 10.1001/jamanetworkopen.2020.28786 PMC7662136

[12] Lee J. Mental health effects of school closures during COVID-19. Lancet Child Adolesc Health. 2020;4(6):421. 32302537 10.1016/S2352-4642(20)30109-7 PMC7156240

[13] Hillis SD, Blenkinsop A, Villaveces A, . COVID-19–associated orphanhood and caregiver death in the US. Pediatrics. 2021;e2021053760. 34620728 10.1542/peds.2021-053760 PMC10896160

[14] Rider EA, Ansari E, Varrin PH, Sparrow J. Mental health and well-being of children and adolescents during the Covid-19 pandemic. BMJ. 2021;374:n1730. 34429302 10.1136/bmj.n1730

[15] Hause AM, Baggs J, Marquez P, . COVID-19 vaccine safety in children aged 5-11 years—US, November 3-December 19, 2021. MMWR Morb Mortal Wkly Rep. 2021;70(5152):1755-1760. 34968370 10.15585/mmwr.mm705152a1 PMC8736274

[16] Bauer BW, Law KC, Rogers ML, Capron DW, Bryan CJ. Editorial overview: analytic and methodological innovations for suicide-focused research. Suicide Life Threat Behav. 2021;51(1):5-7. 33624875 10.1111/sltb.12664

[17] Shim RS. Dismantling structural racism in psychiatry: a path to mental health equity. Am J Psychiatry. 2021;178(7):592-598. 34270343 10.1176/appi.ajp.2021.21060558

[18] Shim RS, Starks SM. COVID-19, structural racism, and mental health inequities: policy implications for an emerging syndemic. Psychiatr Serv. 2021;72(10):1193-1198. 33622042 10.1176/appi.ps.202000725

[19] Bernardini F, Attademo L, Rotter M, Compton MT. Social determinants of mental health as mediators and moderators of the mental health impacts of the COVID-19 pandemic. Psychiatr Serv. 2021;72(5):598-601. 33593101 10.1176/appi.ps.202000393

[20] Lopez L III, Hart LH III, Katz MH. Racial and ethnic health disparities related to COVID-19. JAMA. 2021;325(8):719-720. 33480972 10.1001/jama.2020.26443

[21] Cohn AC, Mahon BE, Walensky RP. One year of COVID-19 vaccines: a shot of hope, a dose of reality. JAMA. 2022;327(2):119-120. 34932067 10.1001/jama.2021.23962

[22] Wrigley-Field E, Garcia S, Leider JP, Van Riper D. COVID-19 mortality at the neighborhood level: racial and ethnic inequalities deepened in Minnesota in 2020. Health Aff (Millwood). 2021;40(10):1644-1653. 34524913 10.1377/hlthaff.2021.00365 PMC8562777

[23] Golberstein E, Wen H, Miller BF. Coronavirus disease 2019 (COVID-19) and mental health for children and adolescents. JAMA Pediatr. 2020;174(9):819-820. 32286618 10.1001/jamapediatrics.2020.1456

[24] Rosenthal DM, Ucci M, Heys M, Hayward A, Lakhanpaul M. Impacts of COVID-19 on vulnerable children in temporary accommodation in the UK. Lancet Public Health. 2020;5(5):e241-e242. 32243776 10.1016/S2468-2667(20)30080-3 PMC7270343

[25] Kidman R, Margolis R, Smith-Greenaway E, Verdery AM. Estimates and projections of COVID-19 and parental death in the US. JAMA Pediatr. 2021;175(7):745-746. 33818598 10.1001/jamapediatrics.2021.0161 PMC8022263

[26] Abrams EM, Szefler SJ. COVID-19 and the impact of social determinants of health. Lancet Respir Med. 2020;8(7):659-661. 32437646 10.1016/S2213-2600(20)30234-4 PMC7234789

[27] Dooley DG, Christakis D. It is time to end the debate over school reopening. JAMA Netw Open. 2021;4(4):e2111125. 33914054 10.1001/jamanetworkopen.2021.11125

[28] Tung EL, Peek ME, Rivas MA, Yang JP, Volerman A. Association of neighborhood disadvantage with racial disparities in COVID-19 positivity in Chicago. Health Aff (Millwood). 2021;40(11):1784-1791. 34724418 10.1377/hlthaff.2021.00695 PMC8975623

[29] Anglin DM, Ereshefsky S, Klaunig MJ, From womb to neighborhood: a racial analysis of social determinants of psychosis in the US. Am J Psychiatry. 2021;178(7):599-610. 33934608 10.1176/appi.ajp.2020.20071091 PMC8655820

[30] Tandon PS, Zhou C, Johnson AM, Gonzalez ES, Kroshus E. Association of children’s physical activity and screen time with mental health during the COVID-19 pandemic. JAMA Netw Open. 2021;4(10):e2127892. 34596669 10.1001/jamanetworkopen.2021.27892 PMC8486978

[31] Pouwels KB, House T, Pritchard E, ; COVID-19 Infection Survey Team. Community prevalence of SARS-CoV-2 in England from April to November, 2020: results from the ONS Coronavirus Infection Survey. Lancet Public Health. 2021;6(1):e30-e38. 33308423 10.1016/S2468-2667(20)30282-6 PMC7786000

[32] Gao S, Rao J, Kang Y, . Association of mobile phone location data indications of travel and stay-at-home mandates with COVID-19 infection rates in the US. JAMA Netw Open. 2020;3(9):e2020485. 32897373 10.1001/jamanetworkopen.2020.20485 PMC7489834

[33] ABCD Research Consortium. Home page. Accessed October 11, 2021. https://abcdstudy.org/

[34] National Archives and Records Administration. Standards for maintaining, collecting, and presenting federal data on race and ethnicity. September 30, 2016. Accessed February 19, 2022. https://www.govinfo.gov/content/pkg/FR-1997-10-30/pdf/97-28653.pdf

[35] Heeringa SG, Berglund PA. A guide for population-based analysis of the Adolescent Brain Cognitive Development (ABCD) study baseline data. bioRxiv. Preprint posted online February 10, 2020. doi:10.1101/2020.02.10.942011

[36] National Institute of Mental Health Data Archive. Adolescent Brain Cognitive Development Study (ABCD) data release: COVID Rapid Response Research (RRR) Survey second data release. Accessed January 27, 2022. https://nda.nih.gov/study.html?id=1225

[37] The New York Times. Covid-19 vaccinations: county and state tracker. January 13, 2022. Accessed January 13, 2022. https://www.nytimes.com/interactive/2020/us/covid-19-vaccine-doses.html

[38] US News & World Report. Who is eligible for a COVID-19 vaccine in your state? May 28, 2021. Accessed January 13, 2022. https://www.usnews.com/news/best-states/articles/covid-19-vaccine-eligibility-by-state

[39] US Centers for Disease Control and Prevention. COVID data tracker. Updated March 20, 2022. Accessed February 27, 2022. https://covid.cdc.gov/covid-data-tracker/#datatracker-home

[40] Rose T, Lindsey MA, Xiao Y, Finigan-Carr NM, Joe S. Mental health and educational experiences among Black youth: a latent class analysis. J Youth Adolesc. 2017;46(11):2321-2340. 28755250 10.1007/s10964-017-0723-3

[41] Cohen S, Kamarck T, Mermelstein R. A global measure of perceived stress. J Health Soc Behav. 1983;24:385-396. 6668417

[42] NIH Toolbox. NIH toolbox scoring and interpretation guide. June 16, 2021. Accessed October 11, 2021. https://nihtoolbox.force.com/s/article/nih-toolbox-scoring-and-interpretation-guide

[43] Github. ABCD-STUDY/geocoding. Accessed January 27, 2022. https://github.com/ABCD-STUDY/geocoding/blob/master/Gen_data_proc.R

[44] Dong E, Du H, Gardner L. An interactive web-based dashboard to track COVID-19 in real time. Lancet Infect Dis. 2020;20(5):533-534. 32087114 10.1016/S1473-3099(20)30120-1 PMC7159018

[45] SafeGraph. Social distancing metrics. Accessed October 11, 2021. https://docs.safegraph.com/docs/social-distancing-metrics

[46] Andersen M, Maclean JC, Pesko MF, Simon KI. Paid sick-leave and physical mobility: evidence from the US during a pandemic. Accessed January 27, 2022. https://EconPapers.repec.org/RePEc:nbr:nberwo:27138

[47] Barch DM, Albaugh MD, Baskin-Sommers A, . Demographic and mental health assessments in the adolescent brain and cognitive development study: updates and age-related trajectories. Dev Cogn Neurosci. 2021;52:101031. 34742018 10.1016/j.dcn.2021.101031 PMC8579129

[48] Funkhouser CJ, Chacko AA, Correa KA, Kaiser AJE, Shankman SA. Unique longitudinal relationships between symptoms of psychopathology in youth: a cross-lagged panel network analysis in the ABCD study. J Child Psychol Psychiatry. 2021;62(2):184-194. 32399985 10.1111/jcpp.13256 PMC7657959

[49] van Dijk MT, Murphy E, Posner JE, Talati A, Weissman MM. Association of multigenerational family history of depression with lifetime depressive and other psychiatric disorders in children: results from the Adolescent Brain Cognitive Development (ABCD) study. JAMA Psychiatry. 2021;78(7):778-787. 33881474 10.1001/jamapsychiatry.2021.0350 PMC8060885

[50] Stoel RD, van den Wittenboer G, Hox J. Analyzing longitudinal data using multilevel regression and latent growth curve analysis. Metodologia de las Ciencias del Comportamiento. 2003;5(1):21-42.

[51] McNeish D, Matta T. Differentiating between mixed-effects and latent-curve approaches to growth modeling. Behav Res Methods. 2018;50(4):1398-1414. 29067672 10.3758/s13428-017-0976-5

[52] Wu YT, Daskalopoulou C, Muniz Terrera G, ; ATHLOS Consortium. Education and wealth inequalities in healthy ageing in 8 harmonised cohorts in the ATHLOS Consortium: a population-based study. Lancet Public Health. 2020;5(7):e386-e394. 32619540 10.1016/S2468-2667(20)30077-3 PMC7739372

[53] Assari S, Boyce S, Bazargan M, Caldwell CH. African Americans’ diminished returns of parental education on adolescents’ depression and suicide in the Adolescent Brain Cognitive Development (ABCD) study. Eur J Investig Health Psychol Educ. 2020;10(2):656-668. 32656052 10.3390/ejihpe10020048 PMC7351357

[54] Harrell FE Jr. Regression Modeling Strategies: With Applications to Linear Models, Logistic and Ordinal Regression, and Survival Analysis. Springer International Publishing; 2015.

[55] West SG, Aiken LS, Krull JL. Experimental personality designs: analyzing categorical by continuous variable interactions. J Pers. 1996;64(1):1-48. 8656311 10.1111/j.1467-6494.1996.tb00813.x

[56] Dong Y, Peng CYJ. Principled missing data methods for researchers. Springerplus. 2013;2(1):222. 23853744 10.1186/2193-1801-2-222 PMC3701793

[57] Raifman J, Bor J, Venkataramani A. Association between receipt of unemployment insurance and food insecurity among people who lost employment during the COVID-19 pandemic in the US. JAMA Netw Open. 2021;4(1):e2035884. 33512519 10.1001/jamanetworkopen.2020.35884 PMC7846943

[58] Chakrabarti S, Hamlet LC, Kaminsky J, Subramanian SV. Association of human mobility restrictions and race/ethnicity-based, sex-based, and income-based factors with inequities in well-being during the COVID-19 pandemic in the US. JAMA Netw Open. 2021;4(4):e217373-e217373. 33825836 10.1001/jamanetworkopen.2021.7373 PMC8027913

[59] Wolfson JA, Leung CW. Food insecurity during COVID-19: an acute crisis with long-term health implications. Am J Public Health. 2020;110(12):1763-1765. 32970451 10.2105/AJPH.2020.305953 PMC7662000

[60] Melchior M, Caspi A, Howard LM, . Mental health context of food insecurity: a representative cohort of families with young children. Pediatrics. 2009;124(4):e564-e572. 19786424 10.1542/peds.2009-0583 PMC4231784

[61] Mozaffarian D, Fleischhacker S, Andrés JR. Prioritizing nutrition security in the US. JAMA. 2021;325(16):1605-1606. 33792612 10.1001/jama.2021.1915

[62] Shachar C, Engel J, Elwyn G. Implications for telehealth in a postpandemic future: regulatory and privacy issues. JAMA. 2020;323(23):2375-2376. 32421170 10.1001/jama.2020.7943

[63] Raza A, Claeson M, Magnusson Hanson L, Westerlund H, Virtanen M, Halonen JI. Home and workplace neighborhood socioeconomic status and behavior-related health: a within-individual analysis. Ann Behav Med. 2021;55(8):779-790. 33580661 10.1093/abm/kaaa116 PMC8311784

[64] Chen YH, Glymour M, Riley A, . Excess mortality associated with the COVID-19 pandemic among Californians 18-65 years of age, by occupational sector and occupation: March through November 2020. PLoS One. 2021;16(6):e0252454. 34086762 10.1371/journal.pone.0252454 PMC8177528

[65] Jay J, Bor J, Nsoesie EO, . Neighbourhood income and physical distancing during the COVID-19 pandemic in the US. Nat Hum Behav. 2020;4(12):1294-1302. 33144713 10.1038/s41562-020-00998-2 PMC8107986

[66] Yuan Y, Deng Z, Chen M, . Changes in mental health and preventive behaviors before and after COVID-19 vaccination: a propensity score matching (PSM) study. Vaccines (Basel). 2021;9(9):1044. 34579281 10.3390/vaccines9091044 PMC8473427

[67] Chen S, Aruldass AR, Cardinal RN. Mental health outcomes after SARS-CoV-2 vaccination in the US: a national cross-sectional study. J Affect Disord. 2022;298(Pt A):396-399. 34774648 10.1016/j.jad.2021.10.134 PMC8580571

[68] Yang LH, Link BG, Susser ES. Examining power relations to understand and address social determinants of vaccine uptake. JAMA Psychiatry. 2021;78(12):1303-1304. 34495319 10.1001/jamapsychiatry.2021.2497

[69] Xiao Y, Lindsey MA. Racial/ethnic, sex, sexual orientation, and socioeconomic disparities in suicidal trajectories and mental health treatment among adolescents transitioning to young adulthood in the USA: a population-based cohort study. Adm Policy Ment Health. 2021;48(5):742-756. 33629220 10.1007/s10488-021-01122-w PMC7904031

[70] Xiao Y, Lindsey MA. Adolescent social networks matter for suicidal trajectories: disparities across race/ethnicity, sex, sexual identity, and socioeconomic status. Psychol Med. 2021;1-12. 33653436 10.1017/S0033291721000465 PMC9772914

[71] Argabright ST, Visoki E, Moore TM, . Association between discrimination stress and suicidality in preadolescent children. J Am Acad Child Adolesc Psychiatry. 2021;S0890-8567(21)01355-1. 34425231 10.1016/j.jaac.2021.08.011 PMC8917360

[72] Stinson EA, Sullivan RM, Peteet BJ, . Longitudinal impact of childhood adversity on early adolescent mental health during the COVID-19 pandemic in the ABCD study cohort: does race or ethnicity moderate findings? Biol Psychiatry Glob Open Sci. 2021;1(4):324-335. 34608463 10.1016/j.bpsgos.2021.08.007 PMC8479935

[73] Office of the Surgeon General. Protecting youth mental health. Accessed January 27, 2022. https://www.hhs.gov/sites/default/files/surgeon-general-youth-mental-health-advisory.pdf

[74] Hansen H, Braslow J, Rohrbaugh RM. From cultural to structural competency—training psychiatry residents to act on social determinants of health and institutional racism. JAMA Psychiatry. 2018;75(2):117-118. 29261827 10.1001/jamapsychiatry.2017.3894

