# Supplementary material for: Association of Social Determinants of Health and Vaccinations With Child Mental Health During the COVID-19 Pandemic in the US
Source: JAMA Psychiatry. 2022 Apr 27;79(6):610–21. doi: 10.1001/jamapsychiatry.2022.0818 (PMC9047762; doi:10.1001/jamapsychiatry.2022.0818)
Supplement: Supplement. — eMethods 1. ABCD Study Design and ABCD COVID Rapid Response Research (RRR) Survey eMethods 2. Additional Data Sources eMethods 3. Consideration of Geographic Heterogeneity in Vaccine eMethods 4. Generalized Linear Mixed Models (GLMM) and Restricted Cubic Splines eMethods 5. Consideration of Clustering and Population-Based Analysis of the Adolescent Brain Cognitive Development (ABCD) Study Data eMethods 6. Sensitivity Analysis eFigure 1. Flowchart of Study Sample Inclusion and Exclusion eFigure 2. Distribution of Race and Ethnicity by Area Deprivation Index (ADI) Deciles, 2020 in Study Sample eFigure 3. Predicted Probabilities of Self-Reported Perceived Stress With 95% CI by Date of Survey Completion among US Children, Based on Restricted Cubic Spline Regression, Stratified by Different Sociodemographic Characteristics eFigure 4. Predicted Probabilities of Self-Reported Perceived Stress With 95% CI by Date of Survey Completion Among US Children, Based on Restricted Cubic Spline Regression, Stratified by Individual-Level Time-Varying Social Determinants of Health (SDoH) Characteristics eFigure 5. Predicted Probabilities of Self-Reported Perceived Stress With 95% CI by Date of Survey Completion Among US Children, Based on Restricted Cubic Spline Regression, Stratified by Community-Level Preexisting and Time-Varying Social Determinants of Health (SDoH) Characteristics eFigure 6. Interaction Effects of Race/Ethnicity and Preexisting Individual SDoH (Household Income) on Child Mental Healtha eFigure 7. Interaction Effects of Race/Ethnicity and Preexisting Individual SDoH (Parent Education) on Child Mental Healtha eFigure 8. Interaction Effects of Race/Ethnicity and Preexisting Individual SDoH (Parent Marital Status) on Child Mental Healtha eFigure 9. Interaction Effects of Race/Ethnicity and Preexisting Structural SDoH (Area Deprivation Index) on Child Mental Healtha eFigure 10. Interaction Effects of Race/Ethnicity and Time-Varying Individual SDoH (Worried About Food) on [file jamapsychiatry-e220818-s001.pdf]

Supplementary Online Content

Xiao Y, Yip PS-F, Pathak J, Mann JJ. Association of social determinants of health and vaccinations with child mental health during the COVID-19 pandemic in the US. *JAMA Psychiatry*. Published online April 27, 2022. doi:10.1001/jamapsychiatry.2022.0818

eMethods 1. ABCD Study Design and ABCD COVID Rapid Response Research (RRR) Survey
eMethods 2. Additional Data Sources
eMethods 3. Consideration of Geographic Heterogeneity in Vaccine
eMethods 4. Generalized Linear Mixed Models (GLMM) and Restricted Cubic Splines
eMethods 5. Consideration of Clustering and Population-Based Analysis of the Adolescent Brain Cognitive Development (ABCD) Study Data
eMethods 6. Sensitivity Analysis
eFigure 1. Flowchart of Study Sample Inclusion and Exclusion
eFigure 2. Distribution of Race and Ethnicity by Area Deprivation Index (ADI) Deciles, 2020 in Study Sample
eFigure 3. Predicted Probabilities of Self-Reported Perceived Stress With 95% CI by Date of Survey Completion among US Children, Based on Restricted Cubic Spline Regression, Stratified by Different Sociodemographic Characteristics
eFigure 4. Predicted Probabilities of Self-Reported Perceived Stress With 95% CI by Date of Survey Completion Among US Children, Based on Restricted Cubic Spline Regression, Stratified by Individual-Level Time-Varying Social Determinants of Health (SDoH) Characteristics
eFigure 5. Predicted Probabilities of Self-Reported Perceived Stress With 95% CI by Date of Survey Completion Among U.S. Children, Based on Restricted Cubic Spline Regression, Stratified by Community-Level Preexisting and Time-Varying Social Determinants of Health (SDoH) Characteristics
eFigure 6. Interaction Effects of Race/Ethnicity and Preexisting Individual SDoH (Household Income) on Child Mental Health[a]
eFigure 7. Interaction Effects of Race/Ethnicity and Preexisting Individual SDoH (Parent Education) on Child Mental Health[a]
eFigure 8. Interaction Effects of Race/Ethnicity and Preexisting Individual SDoH (Parent Marital Status) on Child Mental Health[a]
eFigure 9. Interaction Effects of Race/Ethnicity and Preexisting Structural SDoH (Area Deprivation Index) on Child Mental Health[a]
eFigure 10. Interaction Effects of Race/Ethnicity and Time-Varying Individual SDoH (Worried About Food) on Child Mental Health[a]

© 2022 Xiao Y et al. *JAMA Psychiatry.*

eFigure 11. Interaction Effects of Race/Ethnicity and Time-Varying Individual SDoH (Cannot Afford Food) on Child Mental Health[a]
eFigure 12. Interaction Effects of Race/Ethnicity and Time-Varying Individual SDoH (Unemployment Due to COVID-19 Pandemic on Economy) on Child Mental Health[a]
eFigure 13. Interaction Effects of Race/Ethnicity and Time-Varying Individual SDoH (Disrupted Medical Healthcare Access) on Child Mental Health[a]
eFigure 14. Interaction Effects of Race/Ethnicity and Time-Varying Individual SDoH (Disrupted Mental Health Treatment Access) on Child Mental Health[a]
eFigure 15. Interaction Effects of Race/Ethnicity and Time-Varying Structural SDoH (COVID-19 Case) on Child Mental Health[a]
eFigure 16. Interaction Effects of Race/Ethnicity and Time-Varying Structural SDoH (COVID-19 New Case) on Child Mental Health[a]
eFigure 17. Interaction Effects of Race/Ethnicity and Time-Varying Structural SDoH (COVID-19 Death) on Child Mental Health[a]
eFigure 18. Interaction Effects of Race/Ethnicity and Time-Varying Structural SDoH (COVID-19 New Death) on Child Mental Health[a]
eFigure 19. Interaction Effects of Race/Ethnicity and Time-Varying Structural SDoH (Distance Traveled from Home) on Child Mental Health[a]
eFigure 20. Interaction Effects of Race/Ethnicity and Time-Varying Structural SDoH (Home Dwelling) on Child Mental Health[a]
eFigure 21. Interaction Effects of Race/Ethnicity and Time-Varying Structural SDoH (Device Completely at Home) on Child Mental Health[a]
eFigure 22. Interaction Effects of Race/Ethnicity and Time-Varying Structural SDoH (Full-Time Work) on Child Mental Health[a]
eFigure 23. Interaction Effects of Race/Ethnicity and Time-Varying Structural SDoH (Part-Time Work) on Child Mental Health[a]
eFigure 24. Interaction Effects of Race/Ethnicity and Time-Varying Structural SDoH (Vaccination Eligibility) on Child Mental Health[a]
eFigure 25. Interaction Effects of Race/Ethnicity and Time-Varying Structural SDoH (Vaccination Rates) on Child Mental Health[a]
eTable 1. Area Deprivation Index (ADI) Item and Components
eTable 2. Measures, Variables, and Recoding Mechanisms
eTable 3. Results of Multilevel Generalized Mixed Models on Associations between Preexisting and Time-Varying Social Determinants of Health (SDoH) Factors[a]
eReferences

This supplementary material has been provided by the authors to give readers additional information about their work.

eMethods 1. ABCD Study Design and ABCD COVID Rapid Response Research (RRR) Survey

The Adolescent Brain Cognitive Development Study℠ (ABCD Study®), the largest longitudinal study of brain development and child health in the United States, follows over 10 years 11,878 children recruited from 21 U.S. research sites, recruited at ages 9-10 in 2016-18. In March 2020, when our participants were ages 11- to 13-years-old, the world became affected by the COVID-19 pandemic, leading to an upheaval in the economy and the lives of almost every family. The ABCD Study developed brief surveys sent electronically to all ABCD participants and their participating parent/guardian about the impact of the pandemic on their lives. An overview of the ABCD Study is at https://abcdstudy.org.

The ABCD Research Consortium sent Survey 1 on May 16-22, 2020, Survey 2 on June 24-27, 2020, and Survey 3 on August 3 4-5, 2020. Data from these first three surveys constituted the first data release (http://dx.doi.org/10.15154/1520584). Surveys 4, 5, and 6 were sent on October 8, 2020, December 13, 2020, and March 2, 2021. Surveys 4-6 can be found in the second data release (DOI: 10.15154/1522601). DOIs can be found at https://nda.nih.gov/study.html?&id=1041 and http://dx.doi.org/10.15154/1522601

Two changing log documents were released for the first data release in December 2020 (Surveys #1, 2, and 3) and the second in June 2021 (Surveys #4, 5, and 6).

© 2022 Xiao Y et al. *JAMA Psychiatry.*

eMethods 2. Additional Data Sources

To incorporate individual- and structural-level social determinants of health (SDoH), we assembled a longitudinal dataset, comprising six main components (details in Figure 2, eMethods 1-2): (1) Adolescent Brain Cognitive Development (ABCD) COVID Rapid Response Research (RRR) on child mental health and experiences reported by children and parents/caregivers through six-wave surveys between May 16, 2020 and April 24, 2021;[1,2] (2) ABCD COVID-19 geocoded data on individual-level time-varying SDoH;[2] (3) ABCD child residential history data on structural-level pre-existing SDoH;[3] (4) ABCD baseline children's sociodemographic characteristics (ABCD 4.0 release);[3] (5) Dates where all adults aged 18 years or older were eligible for vaccines, cross-referenced from The New York Times[4] and US News;[5] (6) Centers for Disease Control and Prevention (CDC) COVID-19 Vaccine Tracker.[6]

We linked the following databases step by step:

1. ABCD first release - child report
2. ABCD first release - parent report
3. ABCD second release - child report
4. ABCD second release - child report
5. COVID-19 Geocoded Metrics[a.]
    a. John Hopkins University (JHU) = county level[b.]
    b. SafeGraph = census block level[c.]
6. ABCD Baseline sociodemographic characteristics[d.]
    a. Child age, sex, race, ethnicity
    b. Parent-report annual household income, the highest level of parental education, and parent marital status
7. ABCD Residential History
    a. Including calculations of Area Deprivation Index (ADI)
8. ABCD American Community Survey Post Stratification Weights
    a. Including family ID
9. ABCD Longitudinal Tracking History
    a. Including site ID tracking
10. Vaccination
    a. Dates where all adults aged 18 years or older are eligible for vaccines.[e.]
    b. Centers for Disease Control and Prevention COVID-19 Vaccine Tracker.[f.]

[a.] Geocoded metrics data were geocoded concerning the date that the survey was disseminated to the participants, not the date they returned their surveys.

[b.] COVID-19 prevalence (i.e., case and death counts) were obtained from a publicly available GitHub repository maintained by the Center of Systems Science and Engineering at Johns Hopkins University. Data sources are cited on their README.md found at https://github.com/CSSEGISandData/COVID-19. These data have the county-level resolution. Raw case and death counts (cumulative) were used to calculate new case/death counts and aggregated rolling 7-day averages. Metrics were population adjusted by U.S. Census measures per county: https://www.census.gov/data/datasets/time-series/demo/popest/2010s-counties-total.html

[c.] Geocoded metrics data were geocoded concerning the date that the survey was disseminated to the participants, not the date they returned their surveys. Daily metrics for mobile device behaviors are publicly available by SafeGraph at the census block level from 2019 through Dec 2020. Based on the SafeGraph website description, "This product is delivered daily (3 days delayed from actual). Daily data is available going back to January 1, 2019. We used v2.1 to create the historical data from January 1, 2019 - to December 31, 2019 (the backfill) and the data from May 10, 2020, forwards. The data was generated using a panel of GPS pings from anonymous mobile devices. We determine the common nighttime location of each mobile device over 6 weeks to a Geohash-7 granularity (~153m x ~153m). We call this common nighttime location the device's "home" for ease of reference. We then

aggregate the devices by home census block group and provide the metrics set out below for each census block group." The goal of incorporating the social distancing is to capture the physical mobility of parents/caregivers and children in ABCD. Despite the social distance policies by states, some low-wage essential workers may still need to go to full-time work and not work remotely. "full-time" working behaviors in SafeGraph were used as a proxy for working full-time. We note that this measure may include time spent at school for some individuals. Individuals must be 13 years or older to be included in the SafeGraph sample. Thus, the sample does not include elementary and middle school students, but high school and college students may be included. We acknowledge that we may classify being at school as being at work using our variable definition for this latter group of students.

[d.] Baseline demographic characteristics were reported by the parent/caregiver on the Parent Demographics Survey as described by Barch and colleagues.[7,8] Race and ethnicity were categorized into five categories, as the 'race_ethnicity' variable from the ABCD ACS Post Stratification Weights data (short name: acspsw03) in the ABCD 4.0 Release. The "Other" categories contain answers by parents who chose other racial groups or multiple racial identities.

[e.] Eligibility dates for vaccination among adults. Data were cross-referenced from the New York Times[4] and US News,[5] where exact dates were collected from state and county health departments.

[f.] Centers for Disease Control and Prevention. COVID-19 Vaccinations in the United States, Jurisdiction. Published January 15, 2022. Accessed January 15, 2022. https://data.cdc.gov/Vaccinations/COVID-19-Vaccinations-in-the-United-States-Jurisdi/unsk-b7fc

© 2022 Xiao Y et al. *JAMA Psychiatry.*

eMethods 3. Consideration of Geographic Heterogeneity in Vaccine

Children in the ABCD study were recruited from 21 geographic locations that are nationally distributed and generally to represent the range of demographic and socioeconomic diversity of the U.S. birth cohorts. These 21 study sites cover 17 States in the U.S.

In December 2020, federal regulators gave emergency use authorization to two-dose vaccines developed by Pfizer and Moderna. Regulators authorized Johnson & Johnson's one-dose vaccine in February 2021 but recommended a pause in its use on April 13, 2021, because of reports of blood clots in a small number of patients. As a result, all 50 states paused or recommended that providers pause those vaccinations. The government ended the Johnson & Johnson pause on April 23, 2021, clearing the way for states to resume vaccinations.

On August 12, regulators gave emergency use authorization for people with weakened immune systems to get a third dose of the Pfizer and Moderna vaccines and expanded that authorization in September and October to include booster shots for many Americans.

On August 23, the federal government approved the Pfizer vaccine for those 16 and older, the first full approval of a Covid-19 vaccine in the country.

On November 3, 2021, The F.D.A. approved the Pfizer vaccine for children ages 5 to 11 on October 29.

However, the pace of vaccinations varies across the country. Geographic variation in vaccinations may be influenced by priority groups, vaccine eligibility, types and strengths of vaccine mandate, mistrust in vaccinations, logistical and bureaucratic challenges in distributions and administrations, and social vulnerability. In our main analysis, we chose the dates when all adults were eligible for vaccination as a proxy for the state's vaccine priority and implementation. We further use the C.D.C. state-level vaccination rates reported on April 30, 2021 (the same month as the last interview reported in the ABCD RRR second data release). We divided the original rates by 4 quantiles for comparisons, with higher quantiles representing a greater percentage of adults aged 18 and above fully vaccinated (had the second dose of a vaccine), which is the variable Series_Complete_18PlusPop_Pct in the C.D.C. raw data. We substituted it with percent of people who are fully vaccinated (have a second dose of a two-dose vaccine or one dose of a single-dose vaccine) based on the jurisdiction where the recipient lives (Series_Complete_Pop_Pct), percent of the population with at least one dose based on the jurisdiction where recipient lives (Administered_Dose1_Pop_Pct), Percent of 18+ population with at least one dose based on the jurisdiction where the recipient lives (Administered_Dose1_Recip_18PlusPop_Pct4), and percent of delivered doses per 100,000 census population (Dist_Per_100K). Results were consistent with the main study results. The distributions of states by adult vaccine eligibility and quantiles of fully vaccinated adults rates are below:

**Dates when Adults were Eligible for Vaccine**			**Percent of adults aged 18 and above fully vaccinated**			
Before Apr 2021	Apr 1-14, 2021	After Apr 15, 2021	1st Quantile	2nd Quantile	3rd Quantile	4th Quantile
MN	CO	CA	FL	CA	CO	CT
OK	CT	OR	MO	OK	MI	MD
SC	FL	VA	SC	OR	NY	MN
UT	MD	VT	UT		PA	VT
	MI				VA	WI
	MO					
	NY					
	PA					
	WI					

[e] Dates were cross-referenced from the Department of Health at different study sites.

© 2022 Xiao Y et al. *JAMA Psychiatry.*

eMethods 4. Generalized Linear Mixed Models (GLMM) and Restricted Cubic Splines

1. Generalized linear mixed models

In the generalized linear mixed models (distribution = Gaussian, link = identity), fixed effects incorporate individual pre-existing characteristics (Model 1), individual time-varying characteristics (Model 2), both pre-existing and time-varying factors at the individual level (Model 3), community-level pre-existing characteristics (Model 4), community time-varying characteristics (Model 5), both pre-existing and time-varying factors at the community level (Model 6), and all SDoH factors (Model 7).

Random effects include random intercepts for subject ID and study site. Random effects were restricted to be uncorrelated. We used interview dates, parameterized as the dates since the first day of data collection (May 16, 2020), as the time trend indicator in the random slope (change per survey date). We did not use the survey numbers as the time variable, because as indicated in the first ABCD COVID Rapid Response Release, Surveys 1-3 did not have a link expiration date when distributed. Thus, parent or youth participants might have completed two surveys at once, and/or the dates for the parent and youth surveys could differ. We identified and excluded these out-of-order observations as recommended. We also check the interview_date variable to assess these issues.

Across the four mental health outcomes, we separately estimated the linear and nonlinear (quadratic, cubic) trajectories over time. We determined the final model by assessing the Akaike information criteria [AIC] and Bayesian information criteria [BIC]) and comparing the likelihood ratio between nested models.[9,10] Model fit indices suggest nonlinear (quadratic) trajectories of PSS and NIH-TB Sadness, and linear trajectories of COVID-19 worry and NIH-TB positive affect.

Results of multilevel generalized mixed effect modeling were presented using estimated intercepts (baseline scores) and regression coefficients (β) with 95% CIs.

2. Restricted cubic splines

We modeled PSS trajectories using restricted cubic splines with 95% CIs relative to the median. Knots were chosen using the Harrell method.[11] Cubic splines are preferred over traditional techniques (e.g., linear models, mandatory categorization)[12] in making full use of the data features. Five knots were created based on the suggested percentiles (5, 27.5, 50, 72.5, 95), representing the following dates: May 22 (Day 6), July 1 (Day 67), August 22 (Day 98), December 18 (Day 216), and March 13, 2021 (Day 301). We included a series of interaction terms between SDoH characteristics and the splines for days since May 16, 2020, to determine whether the changes in mental health during COVID-19, differed across SDoH factors.[13] We use post hoc Wald tests to determine whether the differences in child mental health trajectories across SDoH factors over time were significant. We used the *margins* and *xbrcspline* to generate linear predictions of mental health and to generate the differences in the level of mental health indicators, on given survey dates compared to May 16, 2020, respectively.[14]

© 2022 Xiao Y et al. *JAMA Psychiatry.*

eMethods 5. Consideration of Clustering and Population-Based Analysis of the Adolescent Brain Cognitive Development (ABCD) Study Data

ABCD is a longitudinal, observational study of U.S. children, ages 9-10 at baseline, recruited at random from the household populations in defined catchment areas for each of 21 study sites. The 21 geographic locations that comprise the ABCD research sites are nationally distributed and generally represent the range of demographic and socioeconomic diversity of the U.S. birth cohorts that comprise the ABCD study population. The clustering of participants and the potential for selection bias in study site selection and enrollment are features of the ABCD observational study design that are informative for statistical estimation and inference.[15]

In this study, we referred to recommended robust and efficient approaches to consider two important nested nature of ABCD based on our research questions.

1. Clustering and non-independence of observations

Primary analyses employed generalized linear mixed-effects models (GLMM). To adjust for the nested nature, the ABCD study site and participant ID were included as random effects. We did not include family-level random intercepts as our main associations of interest in this study are the long-term individual trajectories and site-level SDoH variables. Besides, trying to include family-random intercepts in this complex model led to overfitting and singularity problems. In a sensitivity analysis, we compare the model fit and results using three-level GLMM, comparable to the current findings.

2. American Community Survey (ACS) propensity weights

We did not use the ACS propensity weights as the purpose of our study is not to obtain estimates of the examined associations that could be generalized as population-representative (i.e., modeling site characteristics, assuming pseudo-randomization of age, sex, race/ethnicity, family type, parental employment status, family size and Census region).[16] Instead, our study aims to take advantage of the multisite nature of the study to examine factors that might contribute to site-specific SDoH effects across the 21 geographic locations and 17 States.

In addition, as indicated in the first and second Release Notes of the Adolescent Brain Cognitive Development Study℠ (ABCD) COVID Rapid Response Research (RRR) Survey data, all Surveys 1-6 were collected through electronic surveys sent to all ABCD participants and their participating parent/guardian. There may also be selection bias of online survey design that cannot be accounted for by the ACS propensity weights.

Therefore, based on our understanding of it, we think using the ACS propensity weighting would reduce the interpretability of our findings.

In a sensitivity analysis, we compare the model fit and results using multilevel GLMM and the design-based weighted regression, results were comparable to the current findings.

© 2022 Xiao Y et al. *JAMA Psychiatry.*

eMethods 6. Sensitivity Analysis
We conducted several sensitivity analyses.

First, we investigated potential nonlinear trajectories by adding quadratic survey dates after the first dissemination to the mixed-effect models. The final model was selected based on AIC and BIC. Nonlinear models with the quadratic terms of time fit better for perceived stress and NIH-TB sadness, whereas linear models better predicted COVID-19 worry and NIH-TB positive affect in our sample.

Second, to investigate the racial and ethnic differences in the associations between SDoH and child mental health, we tested the interaction effects between race and ethnicity and each SDoH indicator. Results were reported in eFigures 5-24.

Third, we divided the study sample randomly, repeated the GLMM in a training sub-sample and validation subsample. Results using the training sample can be replicated in the second half of the data.

Fourth, following the NIH Toolbox® Emotion Batteries Scoring and Interpretation Guide for the iPad[17] 29 we dichotomized the NIH-TB Sadness and Positive Affect T-scores into whether the children warrant heightened surveillance or concern (>=60) or not (<60), and examined models for trajectories of sadness and positive affect assuming a binomial distribution and a logit link function. Again, the main findings were unchanged.

Fifth, for vaccination data, we referred to the CDC state-level vaccination rates reported on April 30, 2021 (the same month as the last interview date reported in the ABCD RRR second data release), matching with the states where the ABCD children were recruited. We used the percent of people 18+ who are fully vaccinated (have second dose of a two-dose vaccine or one dose of a single-dose vaccine) based on the jurisdiction where the recipient lives (variable name: Series_Complete_18PlusPop_Pct) for the main sensitivity analyses. We also replicated the same set of analyses (explained below) with

1. the percent of people who are fully vaccinated (have a second dose of a two-dose vaccine or one dose of a single-dose vaccine) based on the jurisdiction where the recipient lives (variable name: Series_Complete_Pop_Pct),
2. Percent of population with at least one dose based on the jurisdiction where the recipient lives (variable name: Administered_Dose1_Pop_Pct),
3. Percent of 18+ population with at least one dose based on the jurisdiction where the recipient lives (variable name: Administered_Dose1_Recip_18PlusPop_Pct), and
4. Delivered doses per 100,000 census population (variable name: Dist_Per_100K).

We repeated the above analyses using different dates reported on Jan 30, 2021, Feb 30, 2021, March 30, 2021. Results consistently suggested better child mental health trajectories in states with higher vaccination rates. When replacing the vaccination rates with state ID in the fixed-effect in the final Generalized Linear Mixed Models (GLMM) to test whether there are state variations (state itself) in the changes in child mental health during COVID-19, we found children living in VT (β=-0.43 [95% CI, [-0.85, -0.016], P=0.042), MO (β=-0.75 [95% CI, [-1.21, -0.29], P=0.001), MN (β=-0.55 [95% CI, [-0.99, -0.12], P=0.013), and CT (β=-0.59 [95% CI, [-1.10, -0.082], P=0.023) reported lower stress, and children living in CT (β=2.93 [95% CI, [0.24, 5.63], P=0.033), MN (β=3.52 [95% CI, [1.32, 5.73], P=0.002), and MO (β=2.51 [95% CI, [0.18, 4.83], P=0.035)reported greater positive affect.

Lastly, we conducted all analyses among saturated samples without missing SDoH factors and mental health outcomes. Results of the sensitivity analyses suggested that nonresponse bias did not threaten the validity of our findings. Statistical significance was determined by a 2-sided P value <.05. Analyses and visualization were conducted using Stata (version 17), R, and Python 3.9.

© 2022 Xiao Y et al. *JAMA Psychiatry.*

eFigure 1. Flowchart of Study Sample Inclusion and Exclusion

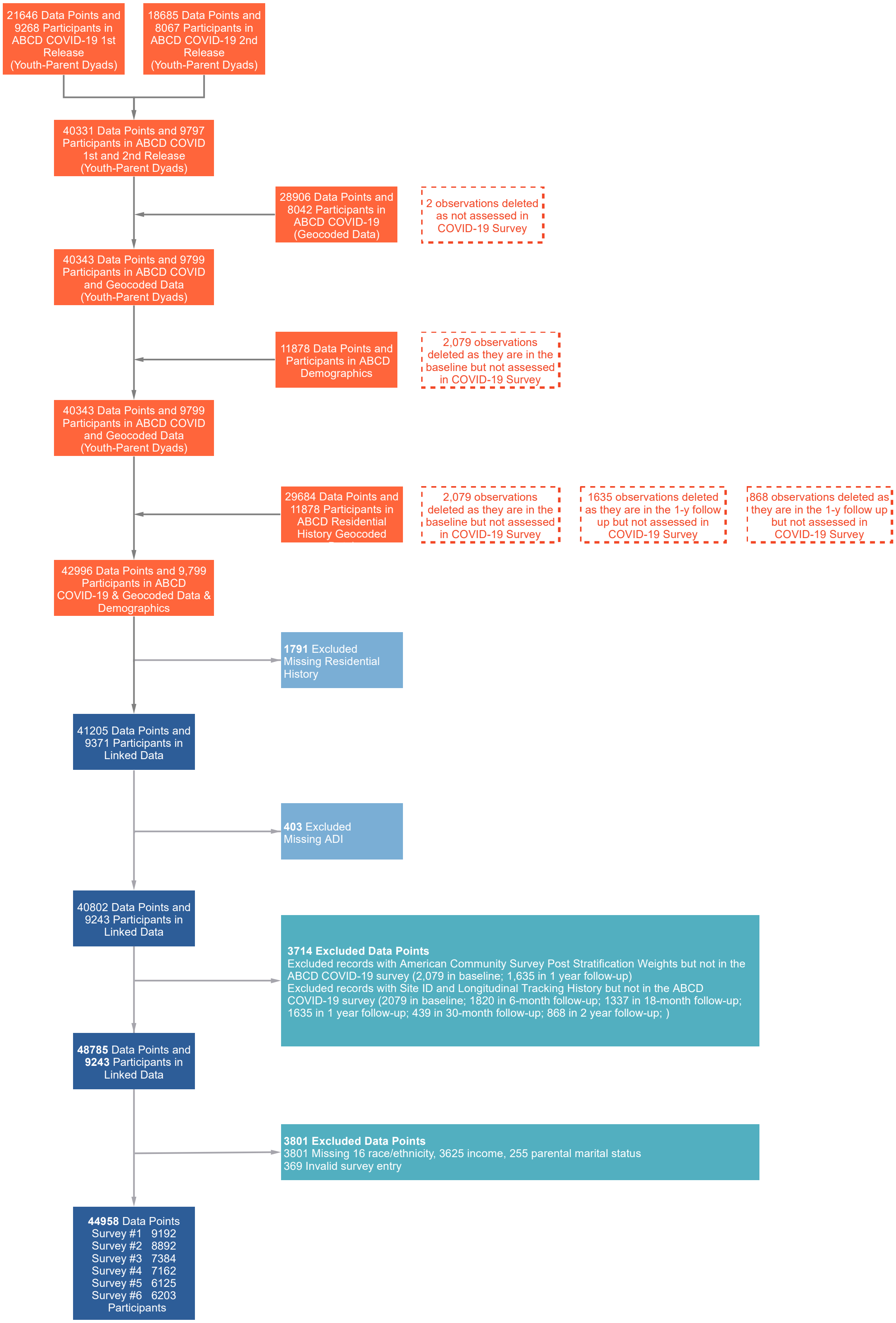

© 2022 Xiao Y et al. *JAMA Psychiatry.*

Participant data were excluded if they reported an invalid primary residential address (*n*=1791), if the ADI score was missing or invalid (*n*=403), and if there were missing data for age, sex, race, ethnicity, household income, parental education, and parental marital status (*n* = 3801). As recommended by the ABCD Release notes,[1] we excluded observations where a participant returned the questionnaire after the dissemination of the subsequent survey (e.g., question in Survey 1 was completed after Survey 2 was completed, *n*=369). For households with siblings, we randomly selected one child in each family to adjust the issues of convergence of random effects. Combining ABCD COVID-19 surveys, main study data, and geocoded information resulted in 52 197 observations across 9799 participants. We included participants with at least three repeated measures for robust estimation of child mental health trajectories and excluded invalid data. The study sample contained 44 958 observations. We kept all records with available information.

© 2022 Xiao Y et al. *JAMA Psychiatry.*

eFigure 2. Distribution of Race and Ethnicity by Area Deprivation Index (ADI) Deciles, 2020 in Study Sample

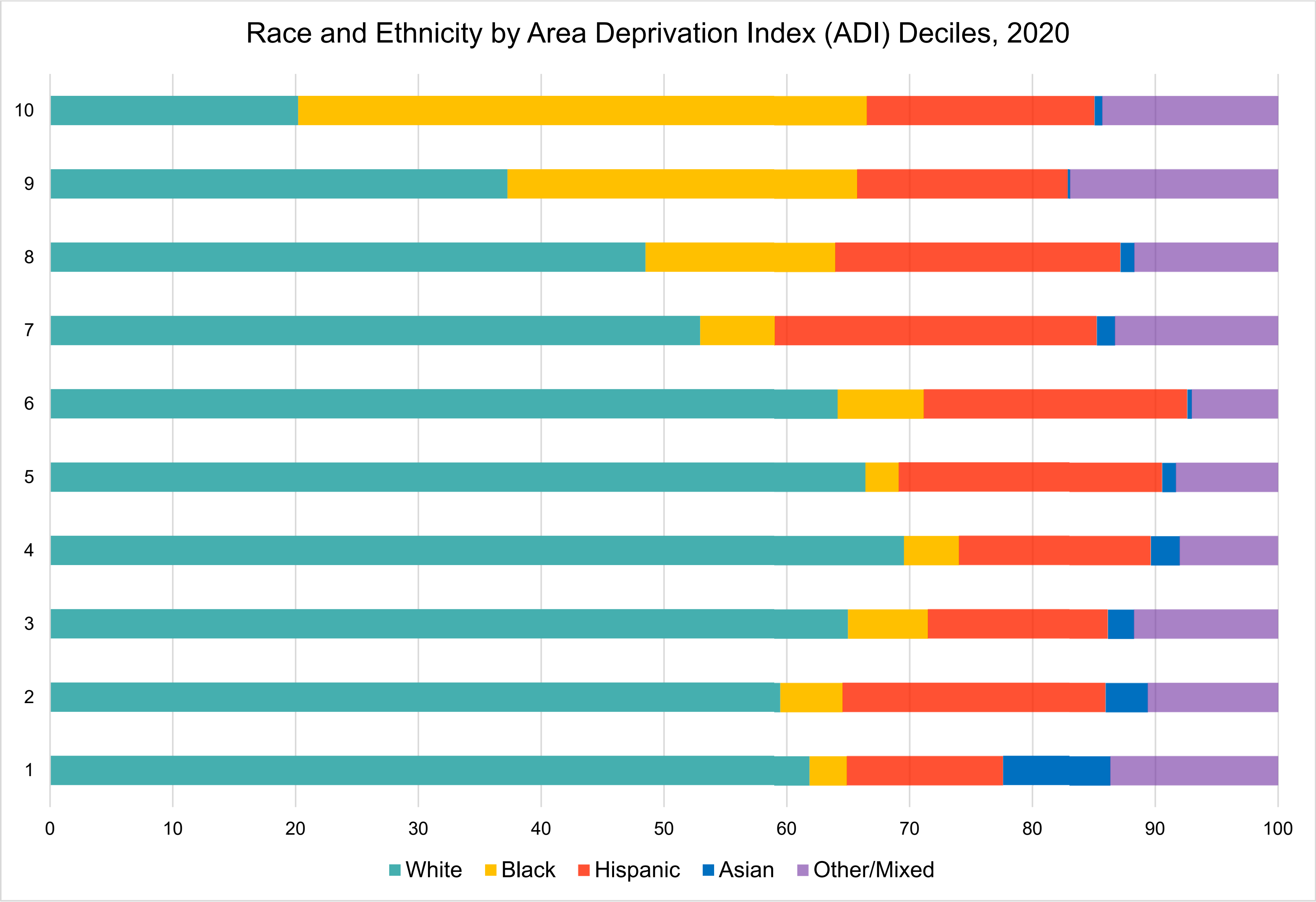

© 2022 Xiao Y et al. *JAMA Psychiatry.*

Note. Race and ethnicity were based on parent-reported demographic characteristics in the ABCD baseline sample (shortname = acspsw03). We used Census-tract-level Area Deprivation Index (ADI) for children's primary residential address at the baseline visit. ADI is a composite weighted metric of 17 neighborhood disadvantage indicators (e.g., poverty rates, unemployment, median family income, low education; see eTable S1). Geocoded ADI in ABCD was based on the 2011–2015 five-year ACS estimates and discretized into national percentiles. We selected ADI as it does not include indirect measures of race and ethnicity in its construction and can limit possible confounding. Based on the percentiles, we categorized ADI into three levels: most, mild, and least deprived areas. The racial and ethnic differences in ADI distribution indicated the need to include both factors in the model.

© 2022 Xiao Y et al. *JAMA Psychiatry.*

eFigure 3. Predicted Probabilities (bold lines) of Self-Reported Perceived Stress with 95% CI (shaded areas) by Date of Survey Completion Among US Children, Based on Restricted Cubic Spline Regression, Stratified by Different Sociodemographic Characteristics

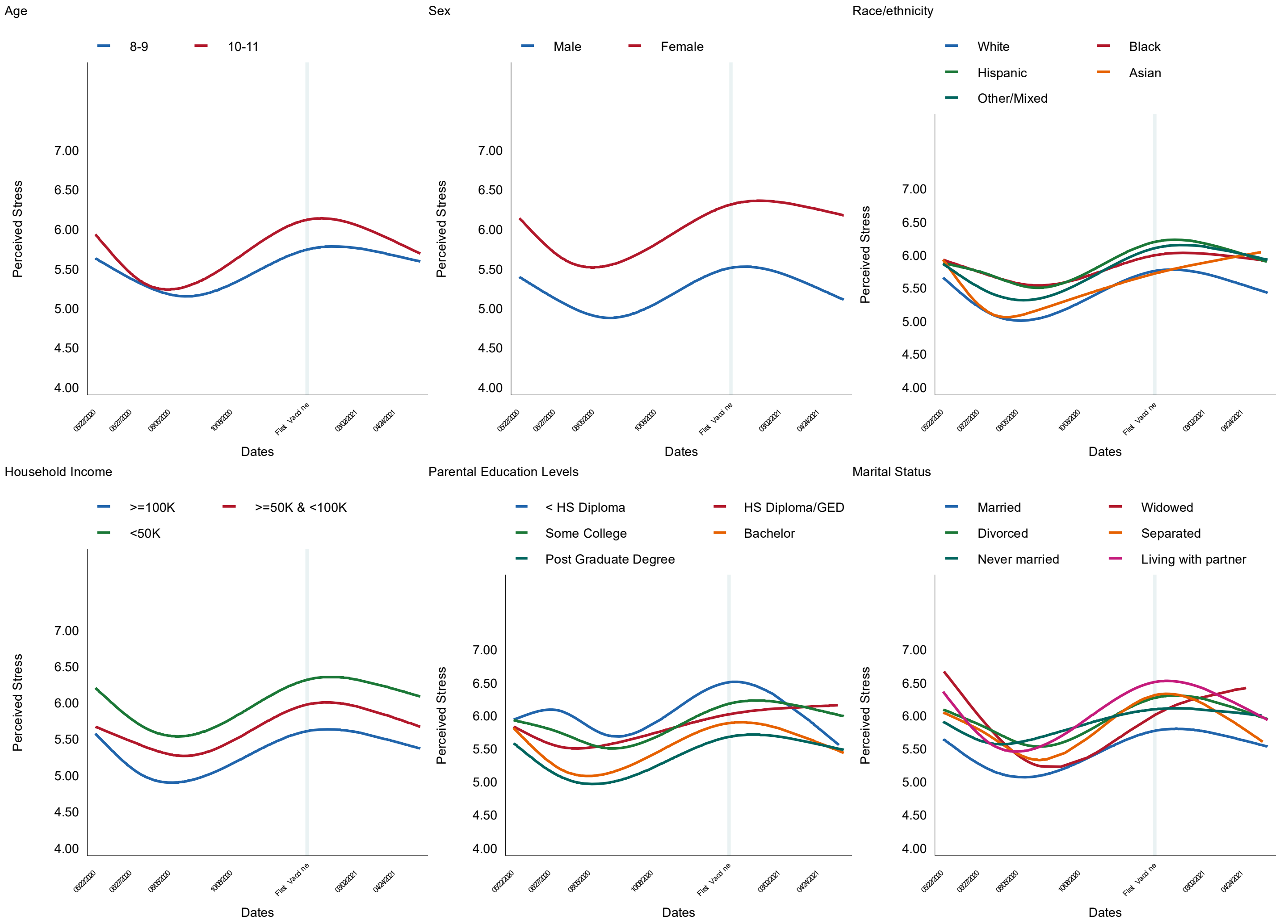

© 2022 Xiao Y et al. *JAMA Psychiatry.*

eFigure 4. Predicted Probabilities (bold lines) of Self-Reported Perceived Stress with 95% CI (shaded areas) by Date of Survey Completion Among US Children, Based on Restricted Cubic Spline Regression, Stratified by Individual-Level Time-Varying Social Determinants of Health (SDoH) Characteristics

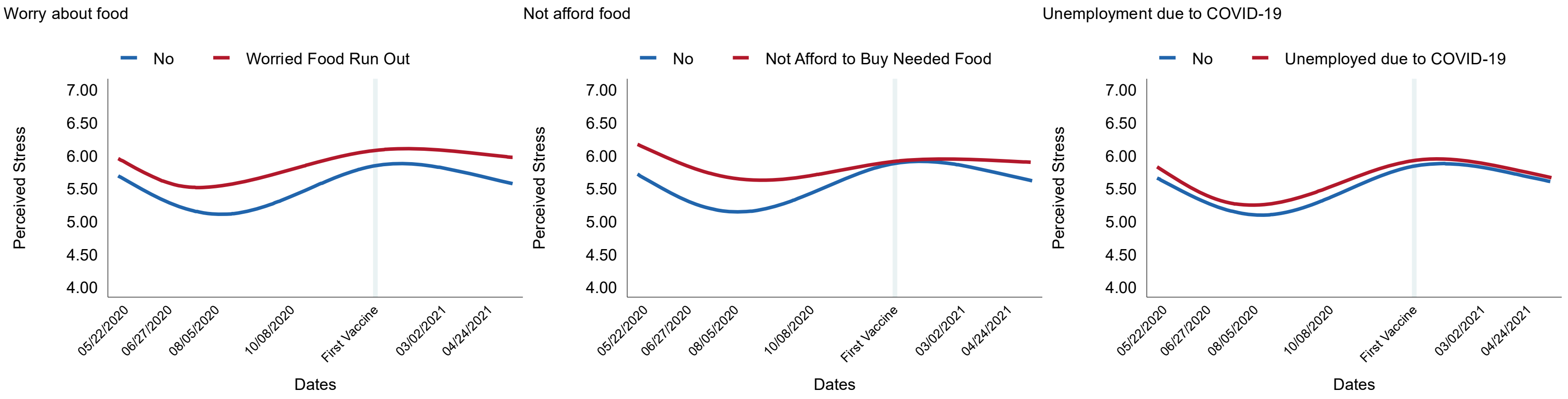

© 2022 Xiao Y et al. *JAMA Psychiatry.*

eFigure 5. Predicted Probabilities (bold lines) of Self-Reported Perceived Stress with 95% CI (shaded areas) by Date of Survey Completion Among US Children, Based on Restricted Cubic Spline Regression, Stratified by Community-Level Preexisting and Time-Varying Social Determinants of Health (SDoH) Characteristics

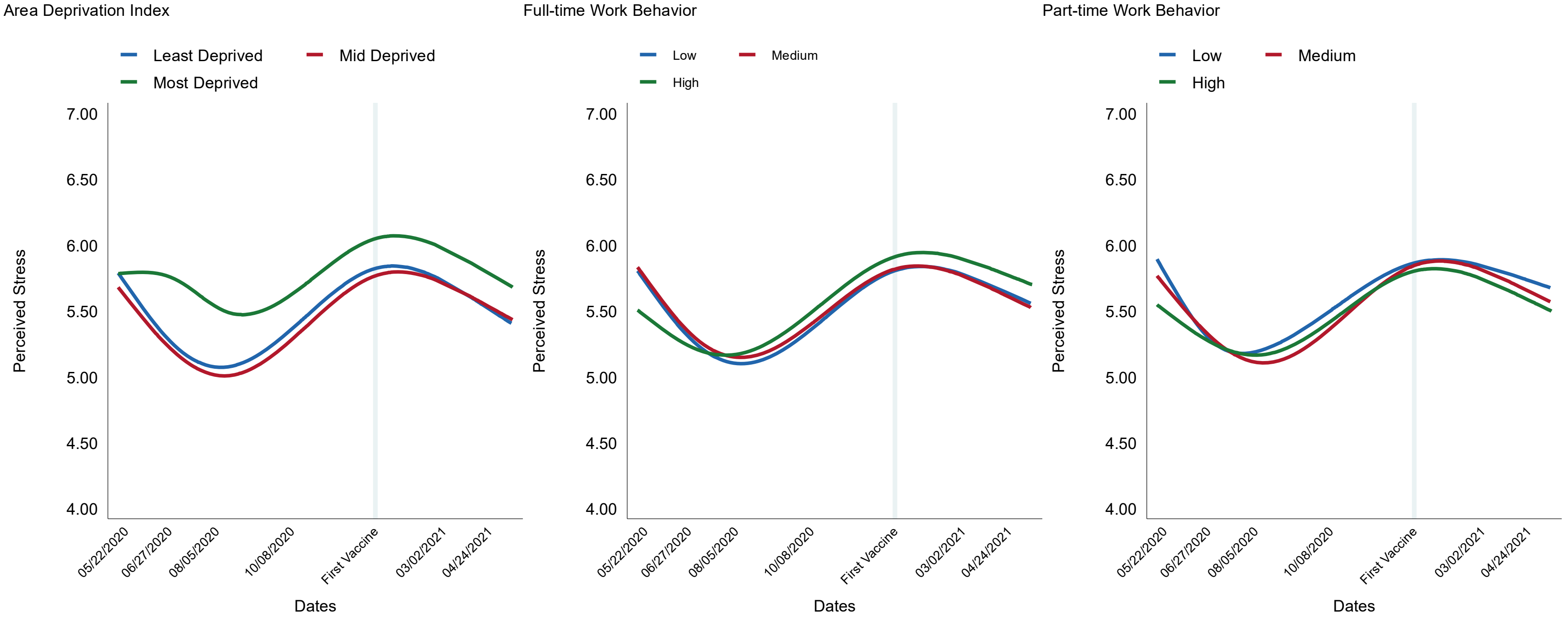

© 2022 Xiao Y et al. *JAMA Psychiatry.*

eFigure 6. Interaction Effects of Race/Ethnicity and Preexisting Individual SDoH (Household Income) on Child Mental Health[a]

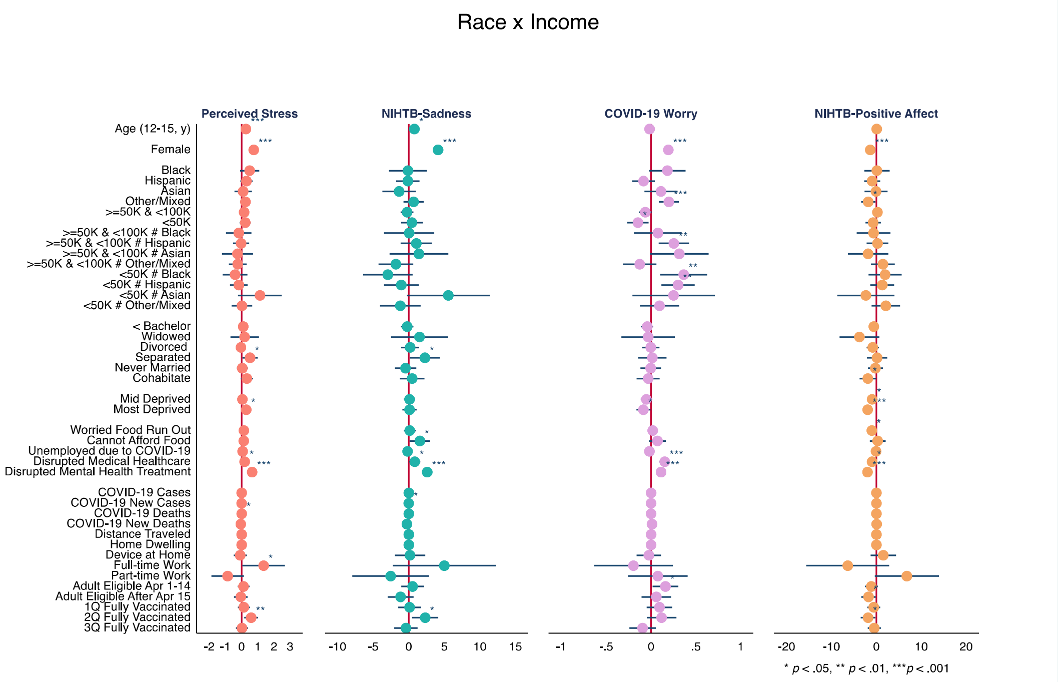

[a] The “Other/Multi-Racial” subcategory was defined when primary caregivers did not identify a specific racial and ethnic group, and primary caregivers were allowed to choose multiple racial subgroups for children.

© 2022 Xiao Y et al. *JAMA Psychiatry.*

eFigure 7. Interaction Effects of Race/Ethnicity and Preexisting Individual SDoH (Parent Education) on Child Mental Health[a]

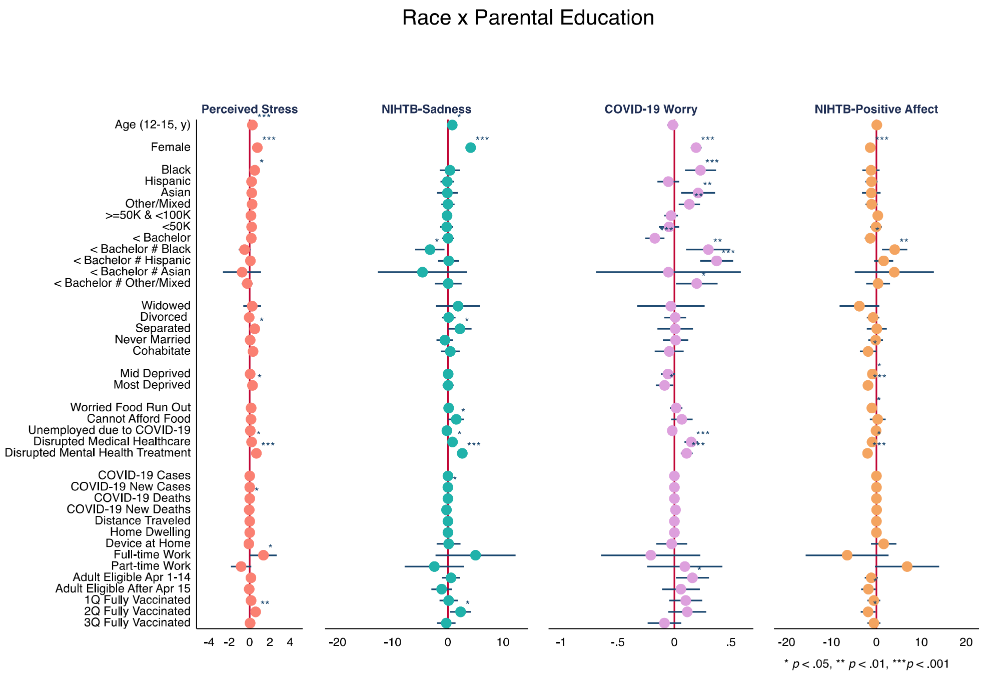

[a] The "Other/Multi-Racial" subcategory was defined when primary caregivers did not identify a specific racial and ethnic group, and primary caregivers were allowed to choose multiple racial subgroups for children.

© 2022 Xiao Y et al. *JAMA Psychiatry.*

eFigure 8. Interaction Effects of Race/Ethnicity and Preexisting Individual SDoH (Parent Marital Status) on Child Mental Health[a]

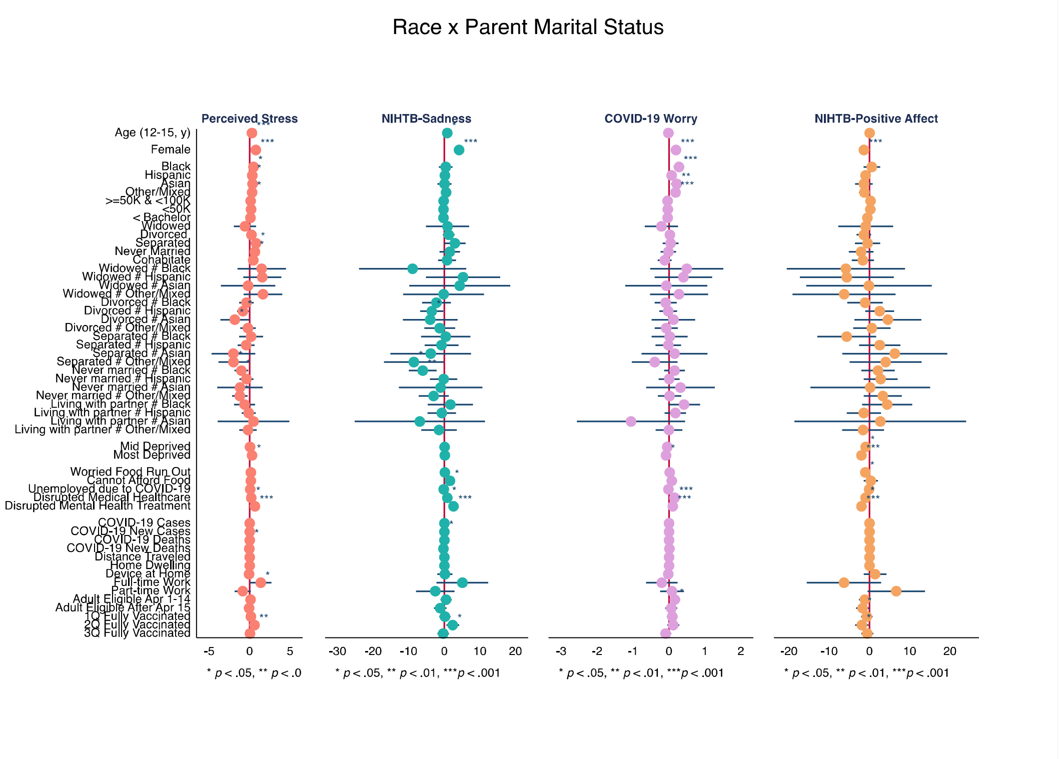

[a] The “Other/Multi-Racial” subcategory was defined when primary caregivers did not identify a specific racial and ethnic group, and primary caregivers were allowed to choose multiple racial subgroups for children.

© 2022 Xiao Y et al. *JAMA Psychiatry.*

eFigure 9. Interaction Effects of Race/Ethnicity and Preexisting Structural SDoH (Area Deprivation Index) on Child Mental Health[a]

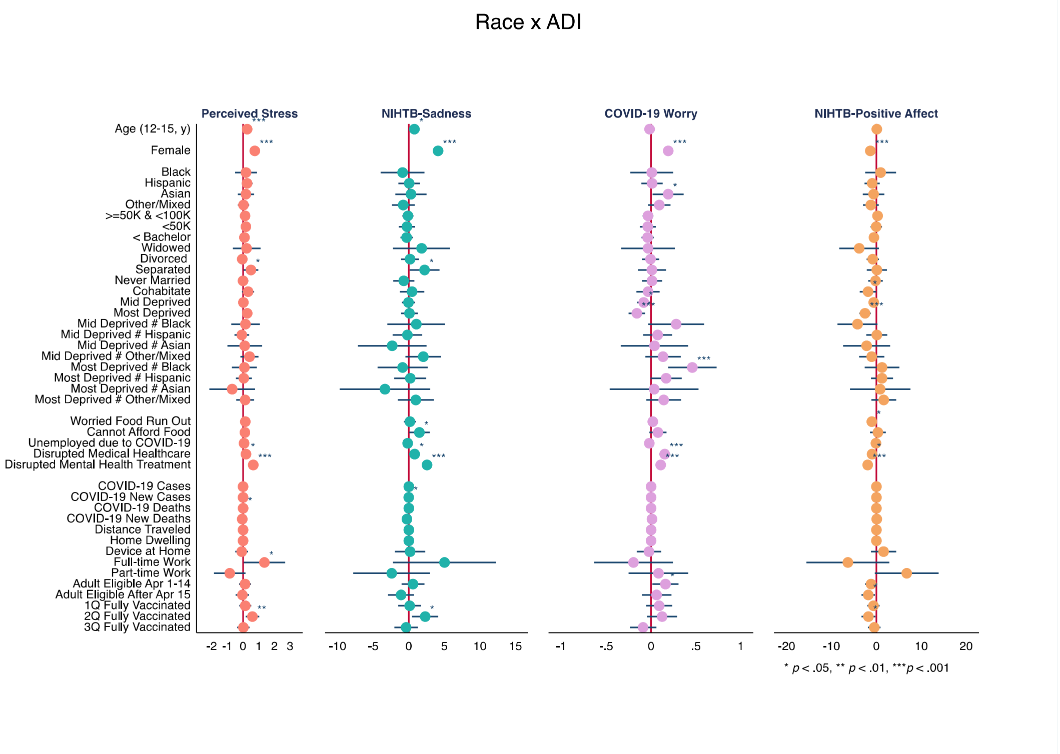

[a] The “Other/Multi-Racial” subcategory was defined when primary caregivers did not identify a specific racial and ethnic group, and primary caregivers were allowed to choose multiple racial subgroups for children.

© 2022 Xiao Y et al. *JAMA Psychiatry.*

eFigure 10. Interaction Effects of Race/Ethnicity and Time-Varying Individual SDoH (Worried about Food) on Child Mental Health[a]

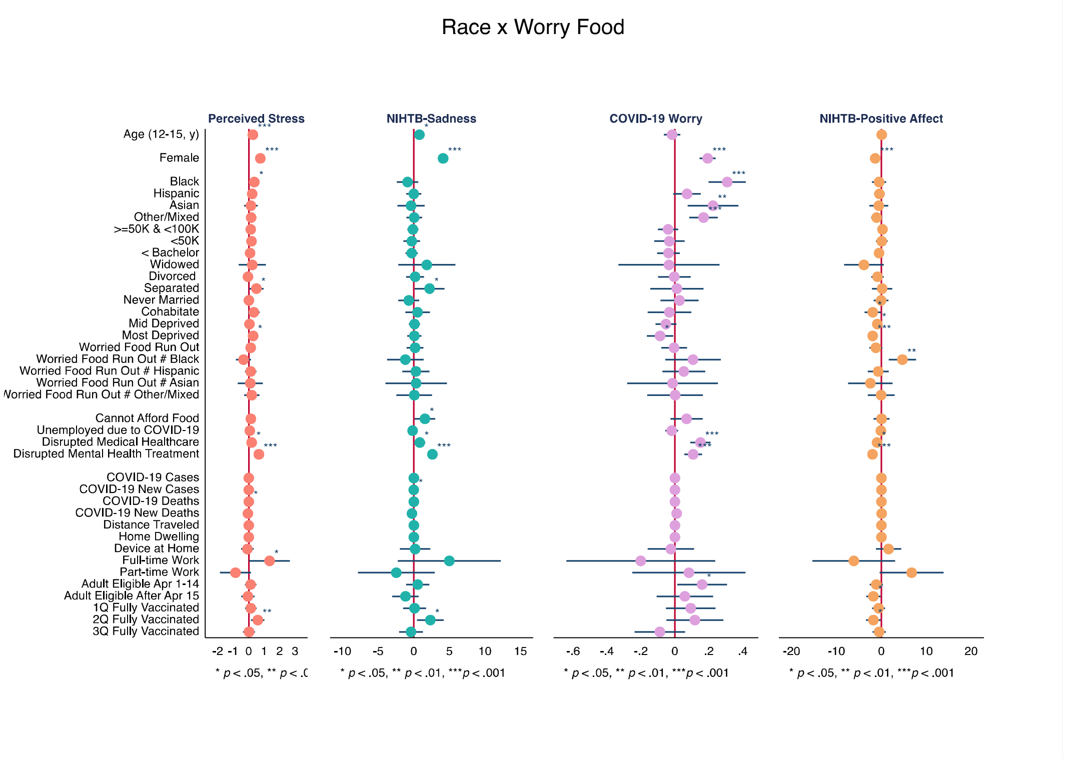

[a] The “Other/Multi-Racial” subcategory was defined when primary caregivers did not identify a specific racial and ethnic group, and primary caregivers were allowed to choose multiple racial subgroups for children.

© 2022 Xiao Y et al. *JAMA Psychiatry.*

eFigure 11. Interaction Effects of Race/Ethnicity and Time-Varying Individual SDoH (Cannot afford Food) on Child Mental Health[a]

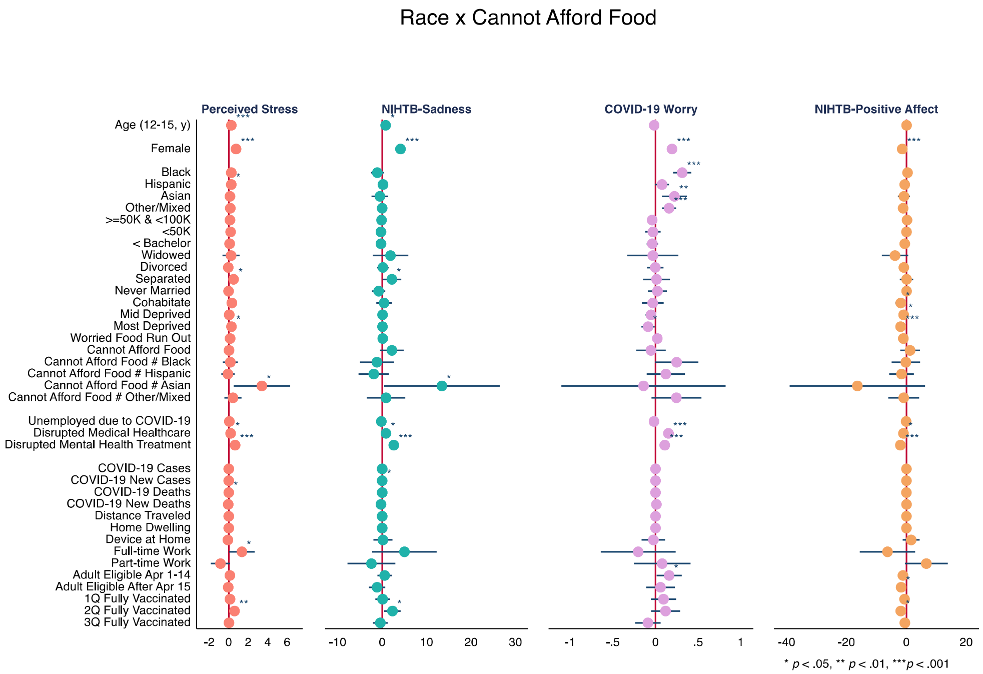

[a] The "Other/Multi-Racial" subcategory was defined when primary caregivers did not identify a specific racial and ethnic group, and primary caregivers were allowed to choose multiple racial subgroups for children.

© 2022 Xiao Y et al. *JAMA Psychiatry.*

eFigure 12. Interaction Effects of Race/Ethnicity and Time-Varying Individual SDoH (Unemployment due to COVID-19 Pandemic on Economy) on Child Mental Health[a]

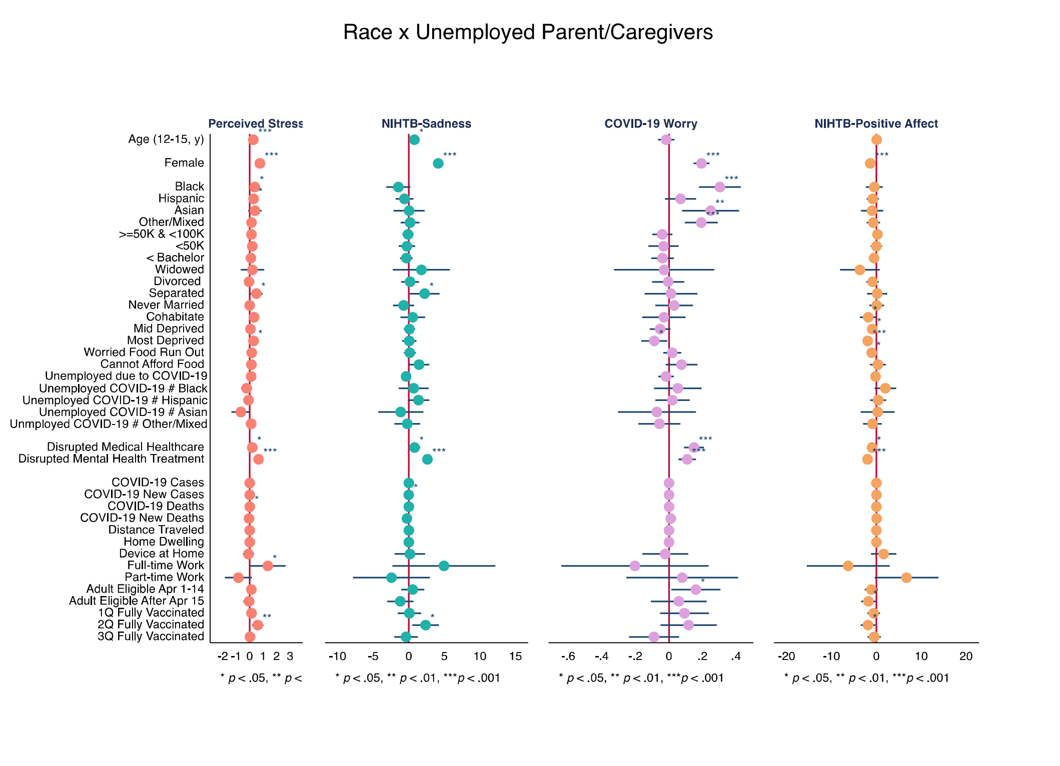

[a] The “Other/Multi-Racial” subcategory was defined when primary caregivers did not identify a specific racial and ethnic group, and primary caregivers were allowed to choose multiple racial subgroups for children.

© 2022 Xiao Y et al. *JAMA Psychiatry.*

eFigure 13. Interaction Effects of Race/Ethnicity and Time-Varying Individual SDoH (Disrupted Medical Healthcare Access) on Child Mental Health[a]

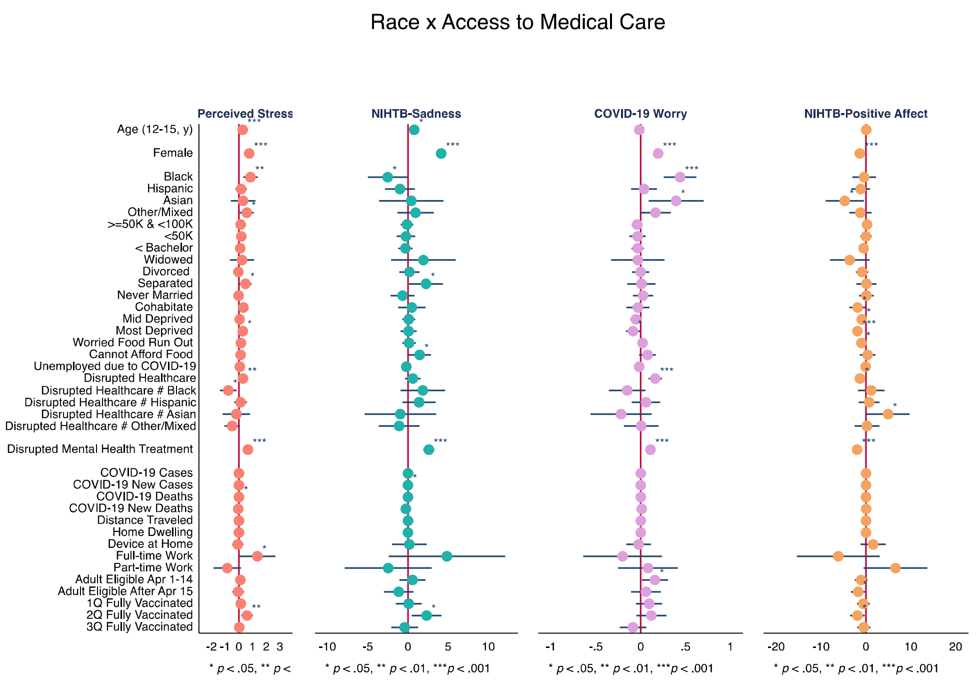

[a] The “Other/Multi-Racial” subcategory was defined when primary caregivers did not identify a specific racial and ethnic group, and primary caregivers were allowed to choose multiple racial subgroups for children.

© 2022 Xiao Y et al. *JAMA Psychiatry.*

eFigure 14. Interaction Effects of Race/Ethnicity and Time-Varying Individual SDoH (Disrupted Mental Health Treatment Access) on Child Mental Health[a]

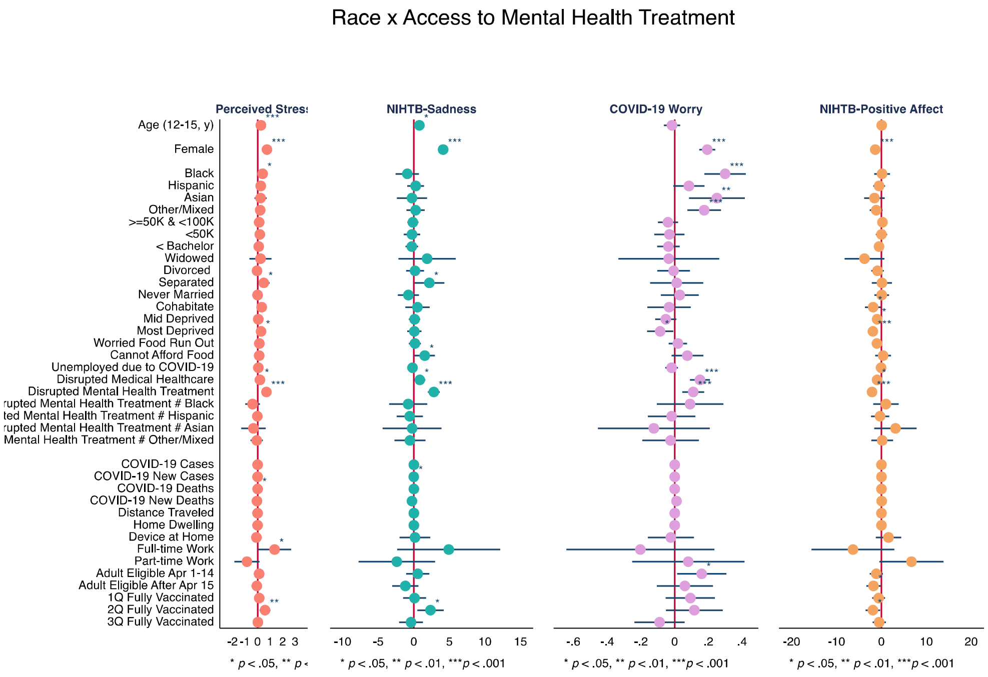

[a] The "Other/Multi-Racial" subcategory was defined when primary caregivers did not identify a specific racial and ethnic group, and primary caregivers were allowed to choose multiple racial subgroups for children.

© 2022 Xiao Y et al. *JAMA Psychiatry.*

eFigure 15. Interaction Effects of Race/Ethnicity and Time-Varying Structural SDoH (COVID-19 Case) on Child Mental Health[a]

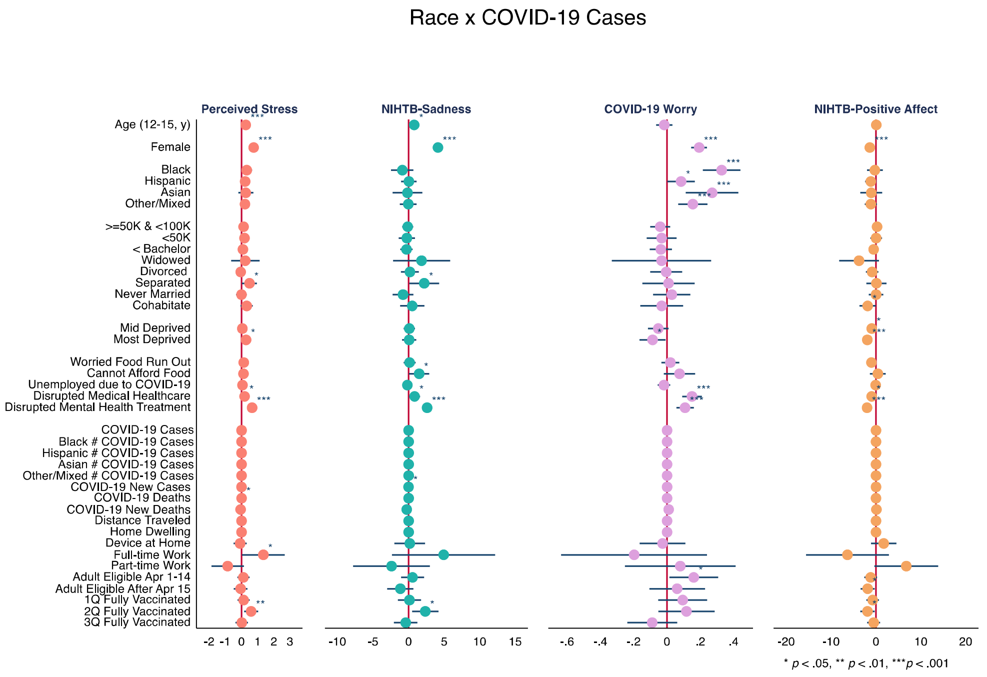

[a] The "Other/Multi-Racial" subcategory was defined when primary caregivers did not identify a specific racial and ethnic group, and primary caregivers were allowed to choose multiple racial subgroups for children.

© 2022 Xiao Y et al. *JAMA Psychiatry.*

eFigure 16. Interaction Effects of Race/Ethnicity and Time-Varying Structural SDoH (COVID-19 New Case) on Child Mental Health[a]

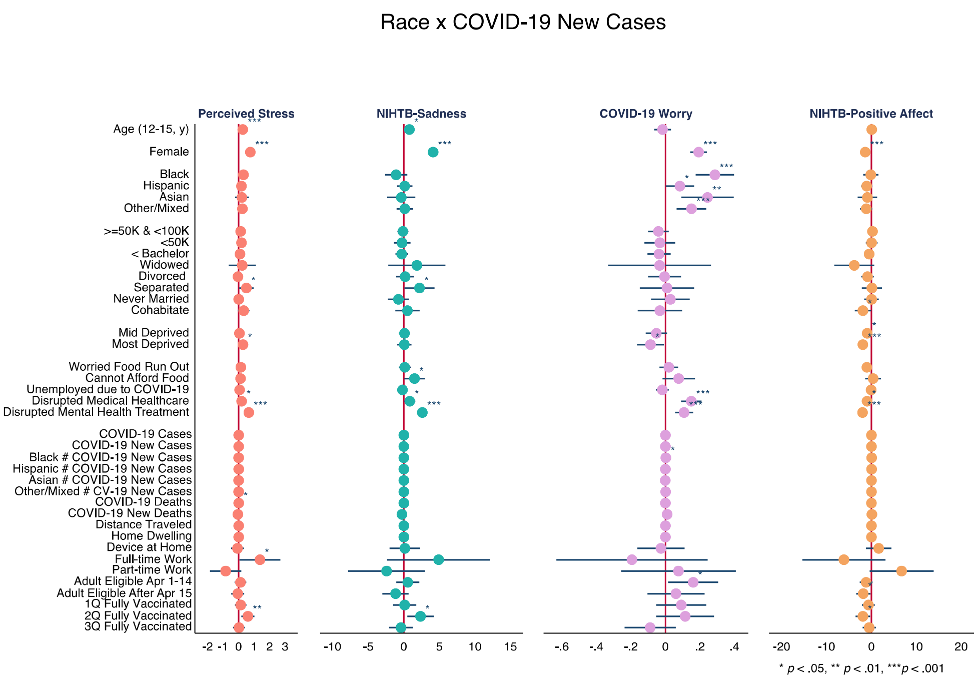

[a] The “Other/Multi-Racial” subcategory was defined when primary caregivers did not identify a specific racial and ethnic group, and primary caregivers were allowed to choose multiple racial subgroups for children.

© 2022 Xiao Y et al. *JAMA Psychiatry.*

eFigure 17. Interaction Effects of Race/Ethnicity and Time-Varying Structural SDoH (COVID-19 Death) on Child Mental Health[a]

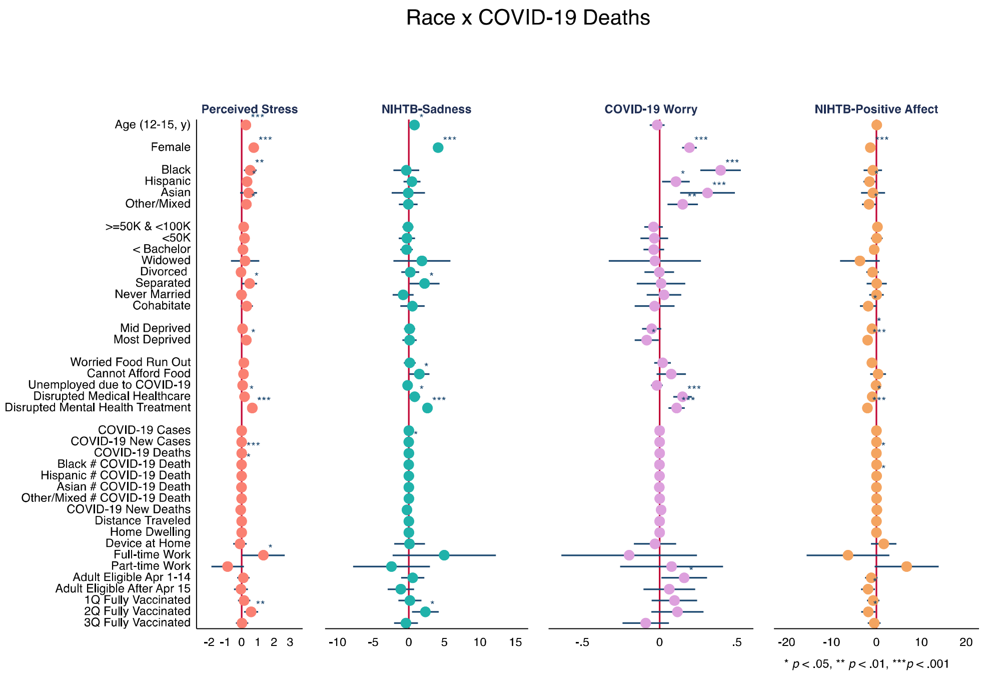

[a] The “Other/Multi-Racial” subcategory was defined when primary caregivers did not identify a specific racial and ethnic group, and primary caregivers were allowed to choose multiple racial subgroups for children.

© 2022 Xiao Y et al. *JAMA Psychiatry.*

eFigure 18. Interaction Effects of Race/Ethnicity and Time-Varying Structural SDoH (COVID-19 New Death) on Child Mental Health[a]

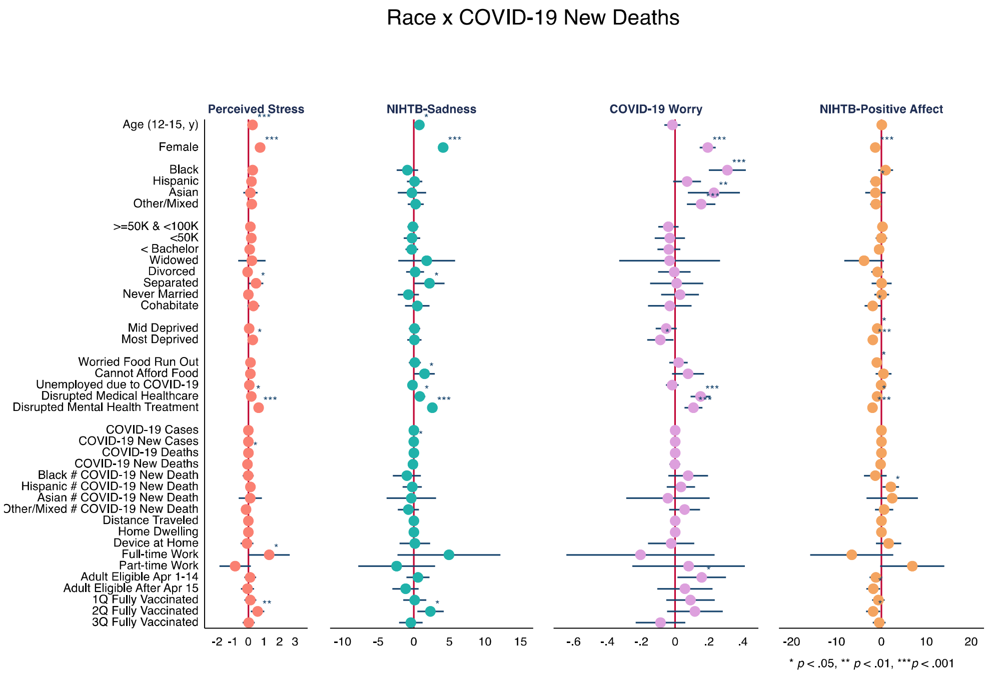

[a] The "Other/Multi-Racial" subcategory was defined when primary caregivers did not identify a specific racial and ethnic group, and primary caregivers were allowed to choose multiple racial subgroups for children.

© 2022 Xiao Y et al. *JAMA Psychiatry.*

eFigure 19. Interaction Effects of Race/Ethnicity and Time-Varying Structural SDoH (Distance Traveled from Home) on Child Mental Health[a]

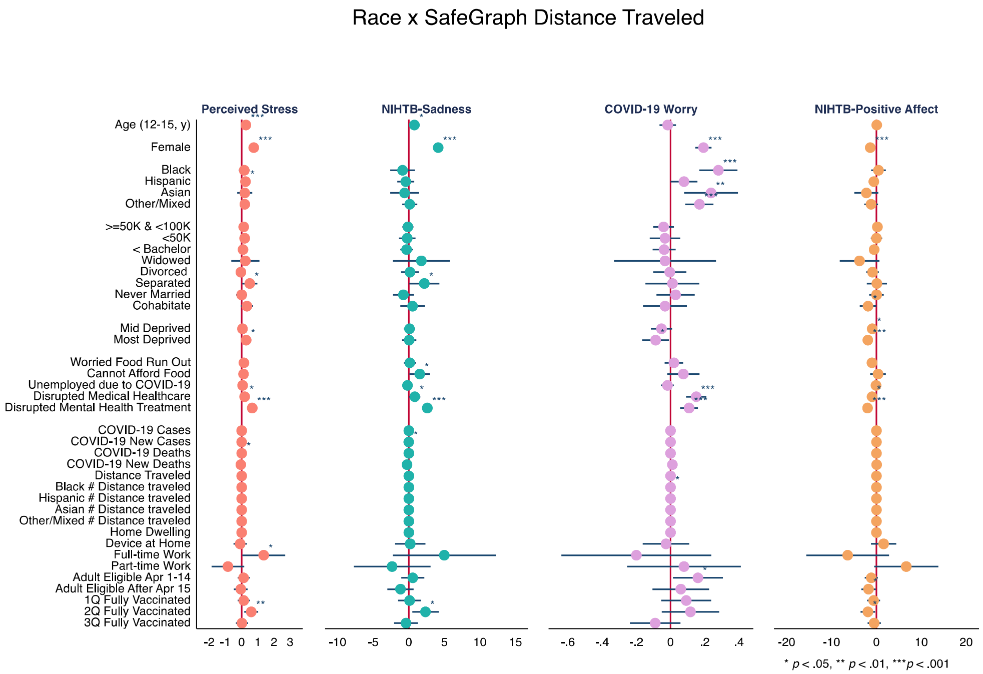

[a] The “Other/Multi-Racial” subcategory was defined when primary caregivers did not identify a specific racial and ethnic group, and primary caregivers were allowed to choose multiple racial subgroups for children.

© 2022 Xiao Y et al. *JAMA Psychiatry.*

eFigure 20. Interaction Effects of Race/Ethnicity and Time-Varying Structural SDoH (Home Dwelling) on Child Mental Health[a]

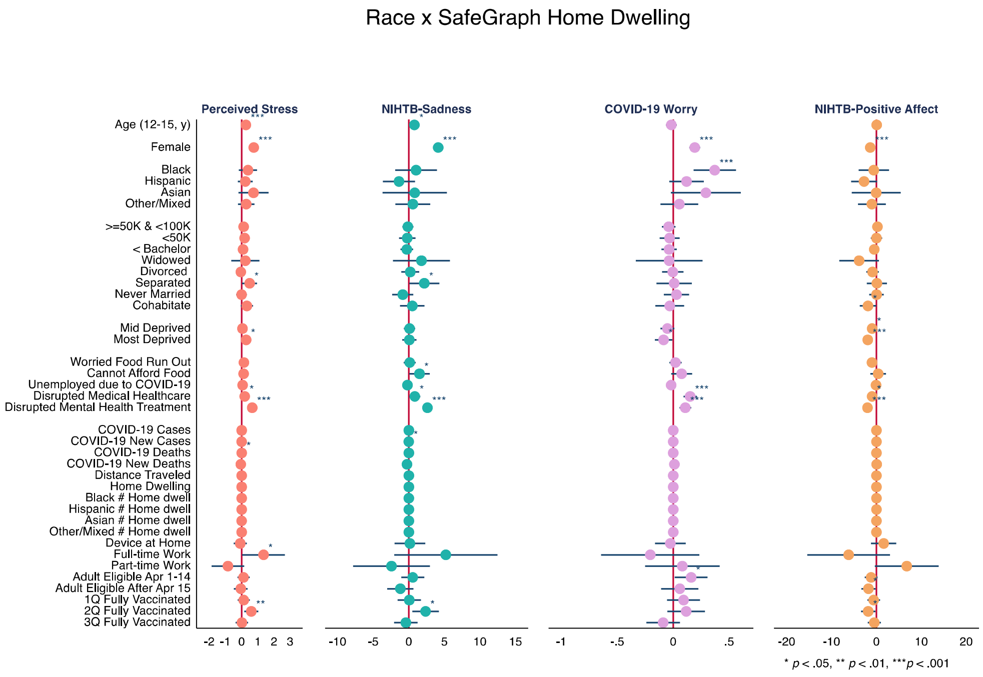

[a] The “Other/Multi-Racial” subcategory was defined when primary caregivers did not identify a specific racial and ethnic group, and primary caregivers were allowed to choose multiple racial subgroups for children.

© 2022 Xiao Y et al. *JAMA Psychiatry.*

eFigure 21. Interaction Effects of Race/Ethnicity and Time-Varying Structural SDoH (Device Completely at Home) on Child Mental Health[a]

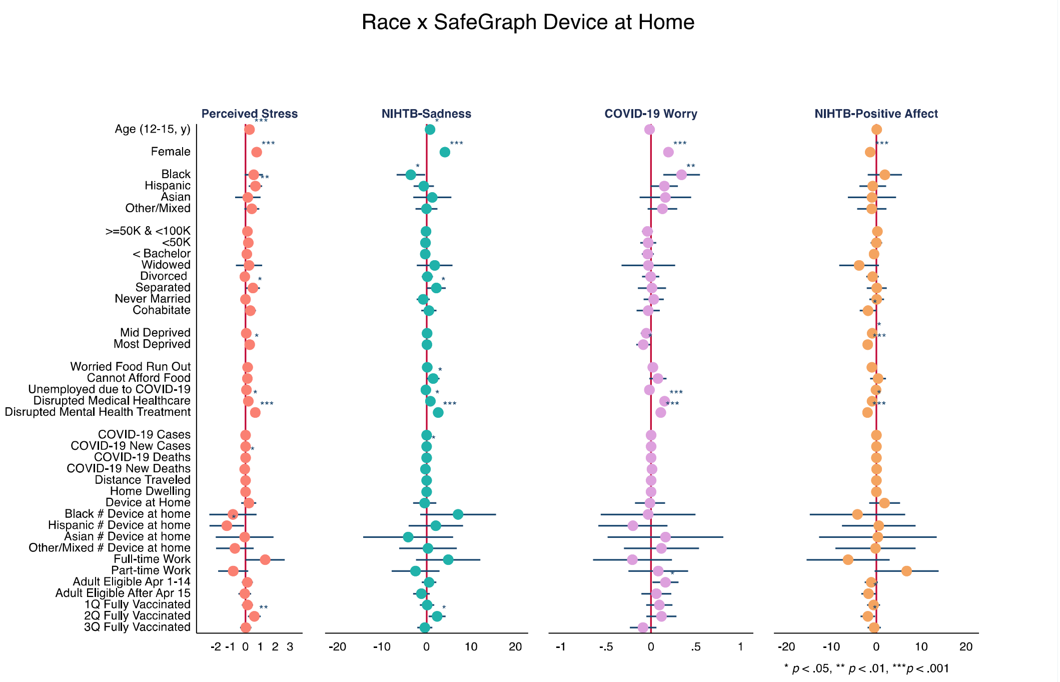

[a] The “Other/Multi-Racial” subcategory was defined when primary caregivers did not identify a specific racial and ethnic group, and primary caregivers were allowed to choose multiple racial subgroups for children.

© 2022 Xiao Y et al. *JAMA Psychiatry.*

eFigure 22. Interaction Effects of Race/Ethnicity and Time-Varying Structural SDoH (Full-Time Work) on Child Mental Health[a]

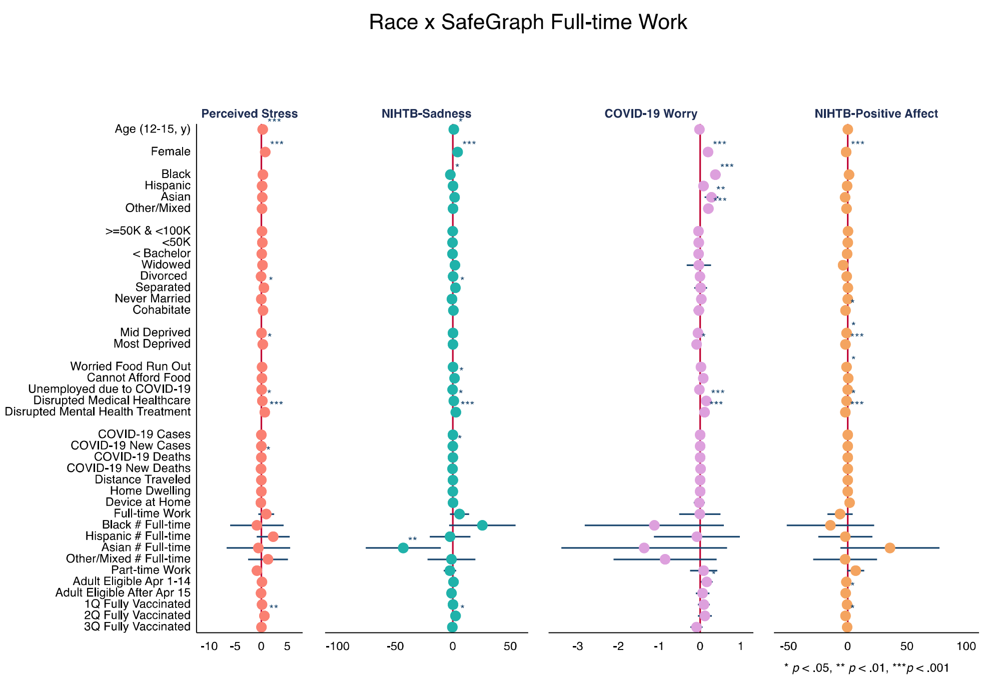

[a] The “Other/Multi-Racial” subcategory was defined when primary caregivers did not identify a specific racial and ethnic group, and primary caregivers were allowed to choose multiple racial subgroups for children.

© 2022 Xiao Y et al. *JAMA Psychiatry.*

eFigure 23. Interaction Effects of Race/Ethnicity and Time-Varying Structural SDoH (Part-Time Work) on Child Mental Health[a]

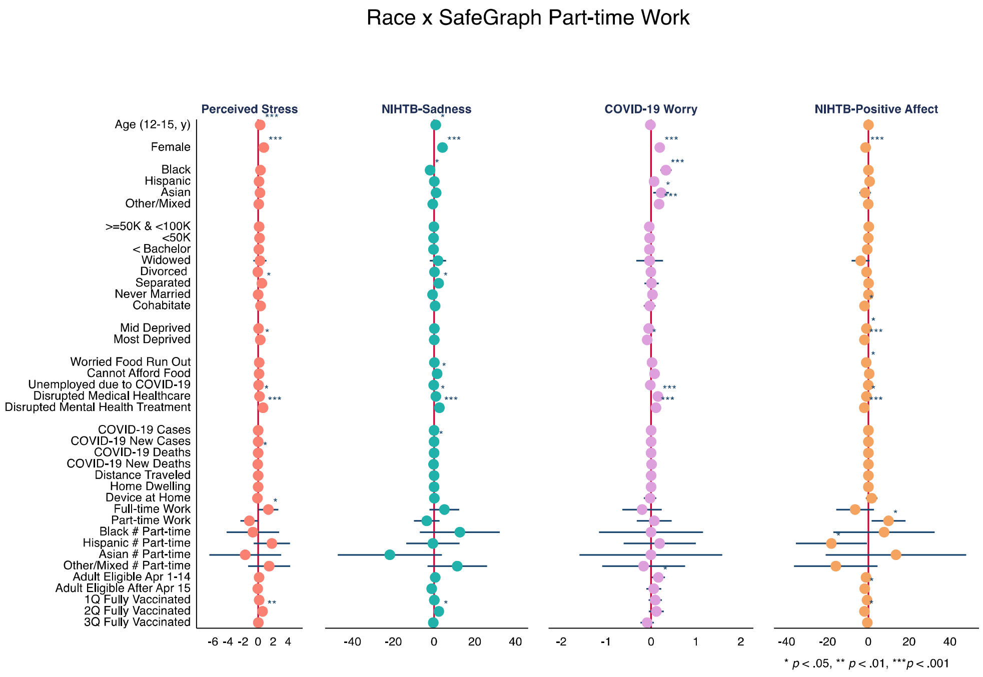

[a] The “Other/Multi-Racial” subcategory was defined when primary caregivers did not identify a specific racial and ethnic group, and primary caregivers were allowed to choose multiple racial subgroups for children.

© 2022 Xiao Y et al. *JAMA Psychiatry.*

eFigure 24. Interaction Effects of Race/Ethnicity and Time-Varying Structural SDoH (Vaccination Eligibility) on Child Mental Health[a]

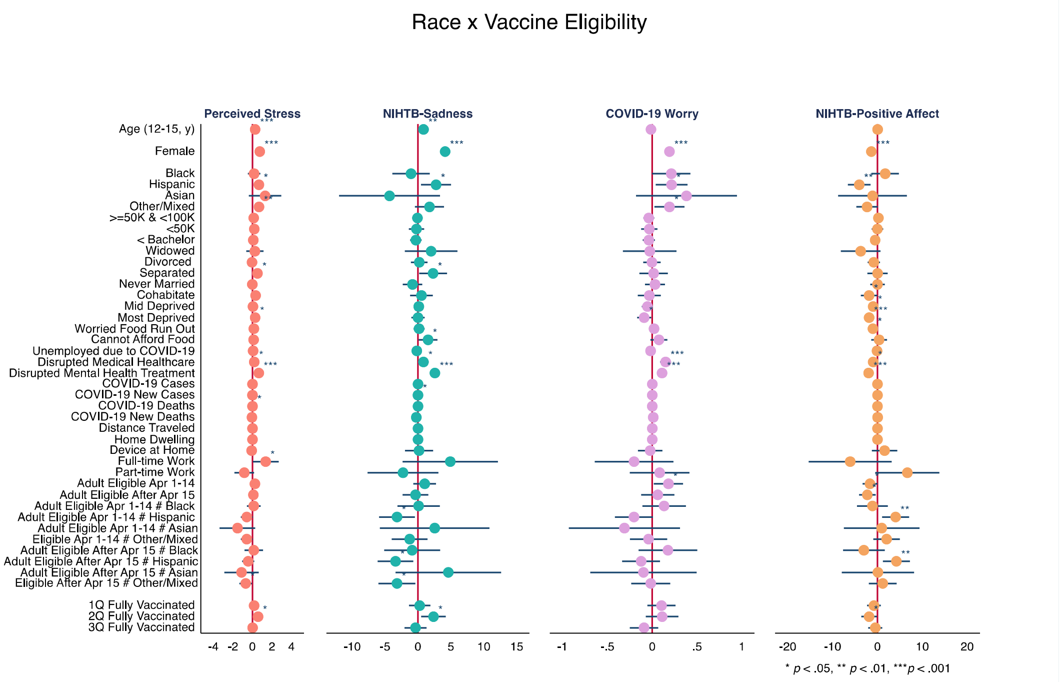

[a] The "Other/Multi-Racial" subcategory was defined when primary caregivers did not identify a specific racial and ethnic group, and primary caregivers were allowed to choose multiple racial subgroups for children.

© 2022 Xiao Y et al. *JAMA Psychiatry.*

eFigure 25. Interaction Effects of Race/Ethnicity and Time-Varying Structural SDoH (Vaccination Rates) on Child Mental Health[a]

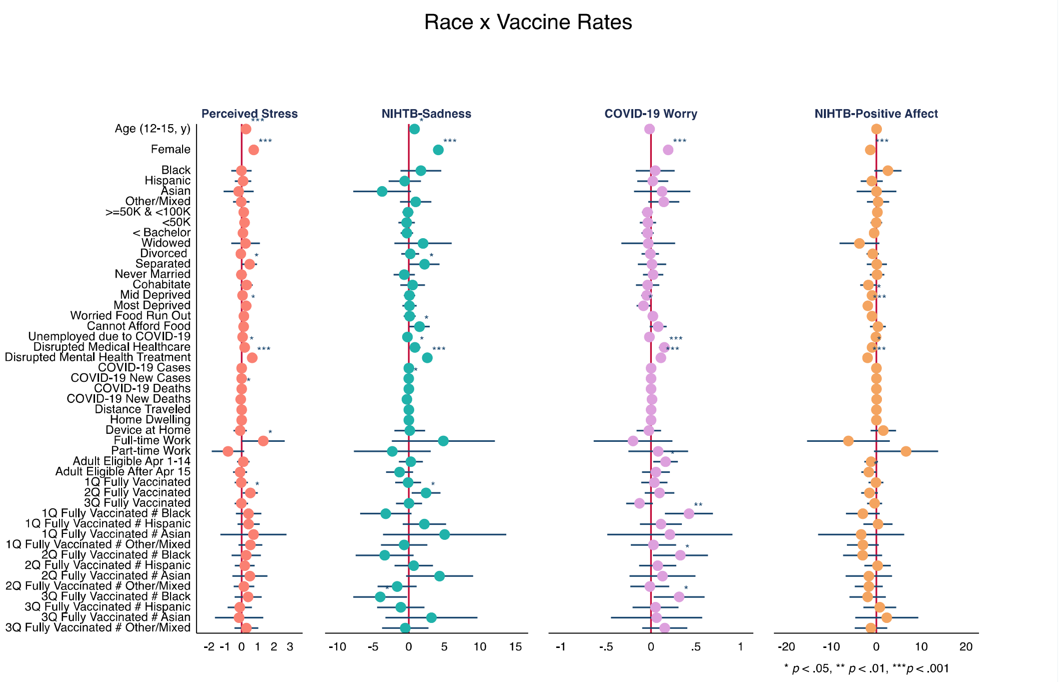

[a] The "Other/Multi-Racial" subcategory was defined when primary caregivers did not identify a specific racial and ethnic group, and primary caregivers were allowed to choose multiple racial subgroups for children.

© 2022 Xiao Y et al. *JAMA Psychiatry.*

eTable 1. Area Deprivation Index (ADI) Item and Components

Item	Area Deprivation Index Components
1	Percentage of population at least 25 years old with less than 9 years of education
2	Percentage of population at least 25 years old with at least a high school diploma
3	Percentage of employed persons at least 16 years old in white-collar occupations
4	Median family income
5	Income disparity
6	Median home value
7	Median gross rent
8	Median monthly mortgage
9	Percentage of owner-occupied housing units (i.e., home ownership rate)
10	Percentage of occupied housing units with more than 1 person per room (i.e., crowding)
11	Percentage of civilian labor force population at least 16 years old who are unemployed (i.e., unemployment rate)
12	Percentage of population below the poverty level
13	Percentage of population below 138% of the poverty threshold
14	Percentage of single-parent households with children under 18 years old
15	Percentage of occupied housing units without a motor vehicle
16	Percentage of occupied housing units without a telephone
17	Percentage of occupied housing units without complete plumbing (log)

© 2022 Xiao Y et al. *JAMA Psychiatry.*

Note. The original proposed Area Deprivation Index (ADI) was contributed by the code for computing and merging ADI (and its national percentile) with ABCD data is available: https://github.com/ABCD-STUDY/geocoding/blob/master/Gen_data_proc.R. Similar to prior studies,[18] we used the ADI, developed by Amy Kind and colleagues,[19] to quantify neighborhood disadvantage and specifically test its association with racial disparities in COVID-19 positivity. The ADI is an ideal measure in analyses of neighborhood effects because it is validated to the census block group level, the closest geographic approximation of a “neighborhood” unit.[19–21] It also captures socioeconomic disadvantage excluding race in its derivation, which enables explicit testing for associations and interactions between the ADI and neighborhood racial composition.

© 2022 Xiao Y et al. *JAMA Psychiatry.*

eTable 2. Measures, Variables, and Recoding Mechanisms

Type	Variable Name	Original Question	Item	Response Categories	Recoding Rules
**Study Design Variables**					
Personal ID	src_subject_id				N/A
Family ID	rel_family_id				N/A
Study Site ID	site_id_l				N/A
**Time Variables**					
Interview Date	interview_date	date of interview, i.e. when non-imaging data was collected	1	recoded into the days since the first date of interview when conducting the cubic spline	N/A
**Outcome: Mental Health**					
Perceived Stress Scale	pstr_confidence_p_cv	In the last month, how often have you felt confident about your ability to handle your personal problems?	4	0 = Never; 1 = Almost Never; 2 = Sometimes; 3 = Fairly often; 4 = Very Often	summed score
	pstr_overcome_p_cv	In the last month, how often have you felt difficulties were piling up so high that you could not overcome them?		0 = Never; 1 = Almost Never; 2 = Sometimes; 3 = Fairly often; 4 = Very Often	
	pstr_unable_control_cv	In the last month, how often have you felt that you were unable to control the important things in your life?		0 = Never; 1 = Almost Never; 2 = Sometimes; 3 = Fairly often; 4 = Very Often	
	pstr_way_p_cv	In the last month, how often have you felt that things were going your way?		0 = Never; 1 = Almost Never; 2 = Sometimes; 3 = Fairly often; 4 =	

© 2022 Xiao Y et al. *JAMA Psychiatry.*

				Very Often	
NIH Toolbox (sadness)	felt_alone_cv	In the past week. . . : I felt alone	8	1 = Never; 2 = Almost Never; 3 = Sometimes; 4 = Often; 5 = Almost Always	T-score
	felt_always_sad	In the past week. . . : I could not stop feeling sad			
	felt_cv	In the past week. . . : I felt like I couldn't do anything right			
	felt_life_went_wrong_cv	In the past week. . . : I felt everything in my life went wrong			
	felt_lonely_cv	In the past week. . . : I felt lonely			
	felt_no_fun_cv	It was hard for me to have fun			
	felt_sad_cv	In the past week. . . : I felt sad			
	felt_unhappy_cv	In the past week. . . : I felt unhappy			
NIH Toolbox (positive affect)	attentive_y_cv	Please rate how each item describes you now or within the past week: I felt attentive (that is, alert or able to pay attention)	9	1 = Not true; 3 = Somewhat true; 5 = Very true; 777 = Refuse to answer	T-score
	calm_y_cv	Please rate how each item describes you now or within the past week. I felt calm			
	concentrate_y_cv	Please rate how each item describes you now or within the past week. I felt able to concentrate			
	confident_y_cv	Please rate how each item describes you now or within the past week. I felt confident			
	delighted_y_cv	Please rate how each item			

© 2022 Xiao Y et al. *JAMA Psychiatry.*

		describes you now or within the past week. I felt delighted			
	ease_y_cv	Please rate how each item describes you now or within the past week. I felt at ease (Definition: relaxed, comfortable)			
	energetic_y_cv	Please rate how each item describes you now or within the past week. I felt energetic			
	enthusiastic_y_cv	Please rate how each item describes you now or within the past week. I felt enthusiastic (Definition: very excited)			
	interested_y_cv	Please rate how each item describes you now or within the past week. I felt interested			
COVID-19 Worry	worry_y_cv	In the past week...How worried have you been about coronavirus (COVID-19)?	1	1 = Not at all; 2 = Slightly; 3 = Moderately; 4 = Very; 5 = Extremely	
**Exposure: Social Determinants of Health (SDoH)**					
Pre-existing [Individual-level]					
Children demographic characteristics	interview_age	Age	1	Computed from months to years of the age, and categorized into two groups: 0 = 8-9; 1 = 10-11	
	sex	Sex	1	0 = Male; 1 = Female	

© 2022 Xiao Y et al. *JAMA Psychiatry.*

	race_ethnicity	Race/ethnicity	1	White<br>Black<br>Hispanic<br>Asian<br>Multi-Racial	The 5-level race/ethnicity variable is consistent with the US Office of Management and Budget (OMB) Standards for presenting data on race and ethnicity.[22] we use the standard 5-level race and ethnicity categorizations to be comparable with previous ABCD studies.[15,23–26]
	demo_comb_income_p	Income	1	<50K<br>>=50K<br>>100K	
Family demographic	demo_prnt_ed_p<br>demo_prtnr_ed_p	Parental education	1	>=Bachelor<br>< Bachelor	

© 2022 Xiao Y et al. *JAMA Psychiatry.*

characteristics	demo_prnt_marital_p	Marital status of the parents	1	Married<br>Widowed<br>Divorced<br>Separated<br>Never married<br>Living with partner	
Pre-existing [Neighborhood-level]					
Area Deprivation Index	reshist_addr1_adi_perc	A multidimensional measure of neighborhood-level socioeconomic (dis)advantage derived from 17 indicators in the Census Bureau's American Community Survey, including education (percent with less than 9 years of education; percent with at least a high school diploma), employment (percent employed in a white-collar occupation; unemployment rate), income (median family income; income disparity; 8 percent below poverty level; percent below 150 percent of poverty level), housing (median home value; median gross rent; median monthly mortgage; home ownership rate), household composition (percent of single parent households), and household resources (percent without a car; percent without a	1	Originally 0-100; recoded to 3 groups by thresholds: 0 = 0-30% least deprived; 1 = 31-60% middle deprived; 2 = 61-100% most deprived	

© 2022 Xiao Y et al. *JAMA Psychiatry.*

		telephone; percent without complete plumbing; percent of housing units with more than one person per room). The ADI measure is constructed by ranking the ADI score from low to high for the nation and grouping the block groups into bins corresponding to each 1 percent range of the ADI score. The national ADI ranks block groups from 1, least disadvantaged, to 100, most disadvantaged in the U.S. In ABCD, the calculation was done using the 2011–2015 five-year ACS estimates.			
Time-varying [Individual-level]					
Food Insecurity	fam_exp1_cv	Were you ever worried about whether your food would run out before you could get more?	1	1 = No; 2 = Yes; 777 = Refuse to answer	
	fam_exp2_cv	Needed food but couldn't afford to buy it or couldn't afford to get out to get it?	1	1 = No; 2 = Yes; 777 = Refuse to answer	
Unemployment	fam_wage_loss_cv	Since January 2020 has anyone in your household lost wages, sales or work due to the impact of coronavirus on		1 = No; 2 = Yes; 777 = Refuse to answer	

© 2022 Xiao Y et al. *JAMA Psychiatry.*

		employment business or the economy?			
Health care and health service access	Mental health treatment access: fam_ht_acc_cv	Please rate how much the coronavirus pandemic has changed your family's life in each of the following ways: mental health treatment access	1	0 = No change.<br>1 = Mild: Appointments moved to telehealth.<br>2 = Moderate: Delays or cancellations in appointments and/or delays in getting prescriptions; changes have minimal impact on health;<br>3 = Severe: Unable to access needed care	0 = No change<br>1 = Mild (appointments moved to telehealth), moderate (delays or cancellations in appointments and/or delays in getting prescriptions; changes have minimal impact on health, and severe (Unable to access needed care) disruptions
	Medical health care access: fam_hc_acc_cv	Please rate how much the coronavirus pandemic has changed your family's life in each of the following ways: medical health care access	1	0 = No change.<br>1 = Mild: Appointments moved to telehealth.<br>2 = Moderate: Delays or cancellations in appointments and/or delays in getting prescriptions; changes have minimal impact on health;	0 = No change<br>1 = Mild (appointments moved to telehealth), moderate (delays or cancellations in appointments

© 2022 Xiao Y et al. *JAMA Psychiatry.*

				3 = Severe: Unable to access needed care	and/or delays in getting prescriptions; changes have minimal impact on health, and severe (Unable to access needed care) disruptions
Time-varying [Neighborhood-level]					
COVID-19 infection and mortality rates	covidgeo_jhu_cases	COVID-19 cases on the interview date	1	Continuous	
	covidgeo_jhu_deaths	COVID-19 new cases on the interview date	1	Continuous	
	covidgeo_jhu_newcases	COVID-19 deaths on the interview date	1	Continuous	
	covidgeo_jhu_newdeaths	COVID-19 new deaths on the interview date	1	Continuous	
Social Mobility	covidgeo_sg_disttravel	Median distance traveled from home (with "home" defined as described above)	1	Continuous	
	covidgeo_sg_homedwell	Average amount of time (in minutes) at home	1	Continuous	
	covidgeo_sg_devathome	Proportion of devices completely at home	1	Continuous	
	covidgeo_sg_ftwork	Proportion of devices exhibiting full-time work behavior	1	Continuous	
	covidgeo_sg_ptwork	Proportion of devices exhibiting part-time work behavior	1	Continuous	

© 2022 Xiao Y et al. *JAMA Psychiatry.*

Vaccination equity	Vaccine eligibility	Dates when all adults became eligible for the vaccine in each state. Reference:	1	Categorical	Before April 2021, April 1-14, and After April 15, 2021
	Vaccine rates	We used the percent of people who are fully vaccinated (have a second dose of a two-dose vaccine or one dose of a single-dose vaccine) based on the jurisdiction where the recipient lives Series_Complete_Pop_Pct for the main sensitivity analyses. We also replicated the same set of analyses (explained below) with 1) the percent of people 18+ who are fully vaccinated (have the second dose of a two-dose vaccine or one dose of a single-dose vaccine) based on the jurisdiction where recipient lives Series_Complete_18PlusPop_ Pct, 2) Percent of population with at lease one dose based on the jurisdiction where recipient lives Administered_Dose1_Pop_Pct 3) Percent of 18+ population with at least one dose based on the jurisdiction where recipient lives Administered_Dose1_Recip_18	1	Categorical	divided into 4 quantiles

© 2022 Xiao Y et al. *JAMA Psychiatry.*

		PlusPop_Pct4) Delivered doses per 100,000 census population Dist_Per_100K			

© 2022 Xiao Y et al. *JAMA Psychiatry.*

eTable 3. Results of Multilevel Generalized Mixed Models on Associations between Preexisting and Time-Varying Social Determinants of Health (SDoH) Factors[a]

	**Perceived Stress**			**NIH-TB Sadness**			**COVID-19 Worry**			**NIH-TB Positive Affect**		
	β	95% CI	*P value*	*β*	95% CI	*P value*	*β*	95% CI	*P value*	*β*	95% CI	*P value*
Demographic Characteristics												
Age (12-15, y)	0.26	[0.12, 0.41]	<.001	0.78	[0.15, 1.41]	.02	-0.02	[-0.07, 0.03]	.50	0.05	[-0.64, 0.74]	.89
Female	0.75	[0.61, 0.89]	<.001	4.12	[3.50, 4.74]	<.001	0.19	[0.14, 0.24]	<.001	-1.39	[-2.07, -0.71]	<.001
Asian	0.15	[-0.28, 0.58]	.49	-0.36	[-2.22, 1.50]	.70	0.22	[0.08, 0.37]	.003	-0.87	[-2.90, 1.15]	.40
Black	0.27	[-0.04, 0.58]	.09	-1.18	[-2.57, 0.22]	.10	0.33	[0.22, 0.43]	<.001	0.46	[-1.03, 1.95]	.55
Hispanic	0.24	[0.01, 0.47]	.039	0.05	[-0.96, 1.06]	.92	0.08	[-0.00, 0.16]	.05	-0.64	[-1.75, 0.46]	.25
Other/Multi-Racial[b]	0.18	[-0.06, 0.42]	.14	0.06	[-1.00, 1.12]	.91	0.17	[0.09, 0.25]	<.001	-1.12	[-2.29, 0.052]	.06
Pre-existing SDoH (individual-level)												
>=50K & <100K	0.13	[-0.05, 0.30]	.16	-0.13	[-0.91, 0.65]	.74	-0.04	[-0.10, 0.02]	.18	0.21	[-0.64, 1.07]	.62
<50K	0.19	[-0.08, 0.45]	.17	-0.26	[-1.42, 0.91]	.67	-0.03	[-0.12, 0.06]	.48	0.00	[-1.29, 1.29]	.99
< Bachelor	0.09	[-0.11, 0.28]	.40	-0.30	[-1.18, 0.58]	.50	-0.04	[-0.10, 0.03]	.28	-0.53	[-1.49, 0.43]	.28
Widowed	0.23	[-0.65, 1.11]	.61	1.82	[-2.19, 5.84]	.37	-0.03	[-0.33, 0.27]	.83	-3.83	[-8.25, 0.60]	.09
Divorced	-0.05	[-0.33, 0.23]	.73	0.17	[-1.09, 1.44]	.79	-0.01	[-0.10, 0.09]	.92	-0.87	[-2.26, 0.52]	.22
Separated	0.50	[0.03, 0.96]	.04	2.20	[0.09, 4.30]	.04	0.01	[-0.15, 0.17]	.91	0.09	[-2.16, 2.34]	.94
Never married	0.00	[-0.33, 0.33]	.99	-0.77	[-2.25, 0.70]	.31	0.03	[-0.08, 0.14]	.61	0.03	[-1.60, 1.66]	.97
Living with partner	0.32	[-0.05, 0.70]	.09	0.50	[-1.22, 2.22]	.57	-0.03	[-0.16, 0.10]	.62	-1.87	[-3.70, -0.046]	.04
Pre-existing SDoH (structural-level)												
Mid deprived	0.05	[-0.13, 0.23]	.60	0.09	[-0.70, 0.89]	.82	-0.05	[-0.12, 0.01]	.09	-0.95	[-1.83, -0.077]	.03
Most deprived	0.28	[0.05, 0.51]	.02	0.06	[-0.94, 1.07]	.90	-0.09	[-0.17, -0.01]	.03	-1.94	[-3.02, -0.85]	<.001
Time-varying SDoH (individual-level)												
Worried Food Run Out	0.14	[-0.02, 0.30]	.09	0.13	[-0.71, 0.97]	.76	0.02	[-0.04, 0.07]	.48	-1.02	[-2.05, 0.016]	.05
Not Afford Needed Food	0.12	[-0.16, 0.41]	.40	1.50	[0.06, 2.93]	.04	0.08	[-0.02, 0.17]	.12	0.34	[-1.42, 2.10]	.71

© 2022 Xiao Y et al. *JAMA Psychiatry.*

Unemployed due to COVID-19	0.06	[-0.05, 0.17]	.29	-0.19	[-0.73, 0.36]	.50	-0.02	[-0.06, 0.02]	.33	-0.10	[-0.74, 0.54]	.76
Disrupted medical health care	0.19	[0.01, 0.36]	.04	0.83	[0.06, 1.61]	.03	0.15	[0.09, 0.21]	<.001	-0.98	[-1.83, -0.13]	.02
Disrupted mental health treatment	0.65	[0.50, 0.81]	<.001	2.60	[1.91, 3.28]	<.001	0.11	[0.06, 0.16]	<.001	-2.00	[-2.75, -1.25]	<.001
Time-varying SDoH (structural-level)												
COVID-19 cases	0.00	[-0.000051, 0.000017]	.32	0.00	[-0.000056, 0.00029]	.19	0.00	[-0.000012, 0.000011]	.95	0.00	[-0.00012, 0.00033]	.37
COVID-19 new cases	0.00	[-0.0029, 0.0012]	.44	-0.01	[-0.023, -0.00037]	.04	0.00	[-0.00070, 0.00066]	.96	0.00	[-0.01, 0.02]	.75
COVID-19 death	0.00	[0.00058, 0.0051]	.01	0.00	[-0.011, 0.010]	.91	0.00	[-0.00089, 0.00065]	.75	-0.01	[-0.02, 0.00]	.33
COVID-19 new death	-0.05	[-0.14, 0.029]	.20	-0.27	[-0.70, 0.17]	.23	0.01	[-0.02, 0.038]	.46	0.04	[-0.60, 0.68]	.91
Distance traveled from home	0.00	[-0.0000057, 0.0000024]	.42	0.00	[-0.000036, 0.0000051]	.14	0.00	[-0.0000012, 0.0000014]	.86	0.00	[-0.000024, 0.000028]	.88
Home dwell	0.00	[-0.000099, 0.00025]	.39	0.00	[-0.0016, 0.00019]	.13	0.00	[-0.000093, 0.000025]	.26	0.00	[-0.00092, 0.0013]	.72
Device completely at home	-0.09	[-0.50, 0.32]	.67	0.15	[-2.00, 2.30]	.89	-0.02	[-0.16, 0.11]	.73	1.55	[-1.27, 4.38]	.28
Full-time work (census block ratio)	1.35	[0.13, 2.67]	.04	4.92	[-2.33, 12.2]	.18	-0.20	[-0.64, 0.24]	.37	-6.35	[-15.6, 2.89]	.18
Part-time work (census block ratio)	-0.85	[-1.86, 0.15]	.10	-2.42	[-7.82, 2.98]	.38	0.08	[-0.25, 0.41]	.64	6.68	[-0.46, 13.8]	.07
Adult Eligible Apr 1-14	0.12	[-0.25, 0.49]	.53	0.55	[-1.08, 2.18]	.51	0.16	[0.01, 0.31]	.03	-1.17	[-2.61, 0.27]	.11
Adult Eligible after Apr 15	-0.06	[-0.48, 0.37]	.80	-1.18	[-3.03, 0.67]	.21	0.06	[-0.11, 0.23]	.48	-1.78	[-3.39, -0.18]	.03
1st Quantile	0.13	[-0.24, 0.51]	.49	0.10	[-1.52, 1.73]	.90	0.09	[-0.05, 0.24]	.21	-0.63	[-2.06, 0.80]	.39
2nd Quantile	0.59	[0.16, 1.02]	.007	2.34	[0.47, 4.21]	.01	0.12	[-0.05, 0.28]	.18	-1.90	[-3.53, -0.26]	.02
3rd Quantile	0.02	[-0.36, 0.40]	.92	-0.41	[-2.07, 1.26]	.63	-0.09	[-0.24, 0.06]	.24	-0.51	[-1.99, 0.96]	.50

[a] Details of each variable measure can be found in eTable 2. We used ABCD Data Release 4.0, which includes the COVID supplemental survey data, consisting of survey responses from ABCD families about the impact of the pandemic on their lives. Surveys were sent electronically to all ABCD participants and their parent/guardian in May, June, August, October, December 2020, and March and May 2021. Also included are COVID-19 geocoded metrics that describe the local environment for each participant, including COVID-19 prevalence and social distancing metrics. Vaccination data are cross-referenced from the CDC Vaccine Tracker, the New York Times, and the US News. Comparisons in

© 2022 Xiao Y et al. *JAMA Psychiatry.*

demographic and pre-existing SDoH were conducted between the ABCD main study sample at baseline (obtained from the ABCD Parent Demographics Survey data) and the ABCD COVID-19 surveys.

[b] This subcategory was listed as "Other/Multi-Racial" but with no specific racial and ethnic group identified, and primary caregivers were allowed to choose multiple racial subgroups for children.

© 2022 Xiao Y et al. *JAMA Psychiatry.*

References

1. ABCD Research Consortium. *COVID Rapid Response Research (RRR) Survey First Data Release*.; 2020. http://dx.doi.org/12805210.15154/1520584
2. ABCD Research Consortium. *COVID Rapid Response Research (RRR) Survey Second Data Release*.; 2021. http://dx.doi.org/10.15154/1522601
3. ABCD Research Consortium. Adolescent Brain Cognitive Development Study (ABCD) - Annual Release 4.0 #1299. doi:10.15154/1523041
4. The New York Times. Covid-19 Vaccinations: County and State Tracker - The New York Times. Published January 13, 2022. Accessed January 13, 2022. https://www.nytimes.com/interactive/2020/us/covid-19-vaccine-doses.html
5. US News. COVID-19 Vaccine Eligibility by State. US News & World Report. Published January 13, 2022. Accessed January 13, 2022. //www.usnews.com/news/best-states/articles/covid-19-vaccine-eligibility-by-state
6. Centers for Disease Control and Prevention. COVID-19 Vaccinations in the United States,Jurisdiction. Published January 15, 2022. Accessed January 15, 2022. https://data.cdc.gov/Vaccinations/COVID-19-Vaccinations-in-the-United-States-Jurisdi/unsk-b7fc
7. Barch DM, Albaugh MD, Baskin-Sommers A, et al. Demographic and mental health assessments in the adolescent brain and cognitive development study: Updates and age-related trajectories. *Dev Cogn Neurosci*. 2021;52:101031. doi:10.1016/j.dcn.2021.101031

© 2022 Xiao Y et al. *JAMA Psychiatry.*

8. Barch DM, Albaugh MD, Avenevoli S, et al. Demographic, physical and mental health assessments in the adolescent brain and cognitive development study: Rationale and description. *Dev Cogn Neurosci*. 2018;32:55-66.
9. McNeish D, Matta T. Differentiating between mixed-effects and latent-curve approaches to growth modeling. *Behav Res Methods*. 2018;50(4):1398-1414. doi:10.3758/s13428-017-0976-5
10. Schwarz G. Estimating the Dimension of a Model. *Ann Stat*. 1978;6(2):461-464.
11. Harrell , FE. *Regression Modeling Strategies: With Applications to Linear Models, Logistic and Ordinal Regression, and Survival Analysis*. Springer International Publishing; 2015. doi:10.1007/978-3-319-19425-7
12. Riehm KE, Holingue C, Smail EJ, et al. Trajectories of mental distress among US adults during the COVID-19 pandemic. *Ann Behav Med*. 2021;55(2):93-102.
13. Grajeda LM, Ivanescu A, Saito M, et al. Modelling subject-specific childhood growth using linear mixed-effect models with cubic regression splines. *Emerg Themes Epidemiol*. 2016;13(1):1. doi:10.1186/s12982-015-0038-3
14. Orsini N. *XBRCSPLINE: Stata Module to Tabulate Differences in Predicted Responses after Restricted Cubic Spline Models*.; 2019. Accessed October 2, 2021. https://econpapers.repec.org/software/bocbocode/s457092.htm
15. Heeringa SG, Berglund PA. A Guide for Population-based Analysis of the Adolescent Brain Cognitive Development (ABCD) Study Baseline Data. *bioRxiv*. Published online January 1, 2020:2020.02.10.942011. doi:10.1101/2020.02.10.942011

© 2022 Xiao Y et al. *JAMA Psychiatry.*

16. Olsen RB, Orr LL, Bell SH, Stuart EA. External validity in policy evaluations that choose sites purposively. *J Policy Anal Manage*. 2013;32(1):107-121.

17. NIH. NIH Toolbox Scoring and Interpretation Guide. Published 2021. Accessed October 11, 2021. https://nihtoolbox.force.com/s/article/nih-toolbox-scoring-and-interpretation-guide

18. Kitchen C, Hatef E, Chang HY, Weiner JP, Kharrazi H. Assessing the association between area deprivation index on COVID-19 prevalence: a contrast between rural and urban U.S. jurisdictions. *AIMS Public Health*. 2021;8(3):519-530. doi:10.3934/publichealth.2021042

19. Kind AJH, Buckingham WR. Making Neighborhood-Disadvantage Metrics Accessible — The Neighborhood Atlas. *N Engl J Med*. 2018;378(26):2456-2458. doi:10.1056/NEJMp1802313

20. Kind AJH, Jencks S, Brock J, et al. Neighborhood Socioeconomic Disadvantage and 30-Day Rehospitalization: A Retrospective Cohort Study. *Ann Intern Med*. 2014;161(11):765. doi:10.7326/M13-2946

21. Chamberlain AM, Finney Rutten LJ, Wilson PM, et al. Neighborhood socioeconomic disadvantage is associated with multimorbidity in a geographically-defined community. *BMC Public Health*. 2020;20(1):13. doi:10.1186/s12889-019-8123-0

22. Office of Management and Budget. Standards for maintaining, collecting, and presenting federal data on race and ethnicity. Federal Register. Published 1997. Accessed February 19, 2022. https://www.govinfo.gov/content/pkg/FR-1997-10-30/pdf/97-28653.pdf

© 2022 Xiao Y et al. *JAMA Psychiatry.*

23. Assari S, Boyce S, Akhlaghipour G, Bazargan M, Caldwell CH. Reward Responsiveness in the Adolescent Brain Cognitive Development (ABCD) Study: African Americans' Diminished Returns of Parental Education. *Brain Sci*. 2020;10(6):391. doi:10.3390/brainsci10060391

24. Assari S. American Children's Screen Time: Diminished Returns of Household Income in Black Families. *Inf Basel*. 2020;11(11). doi:10.3390/info11110538

25. Assari S. Parental Education, Household Income, and Cortical Surface Area among 9–10 Years Old Children: Minorities' Diminished Returns. *Brain Sci*. 2020;10(12):956. doi:10.3390/brainsci10120956

26. Assari S. Multiplicative Effects of Social and Psychological Risk Factors on College Students' Suicidal Behaviors. *Brain Sci*. 2018;8(5). doi:10.3390/brainsci8050091

© 2022 Xiao Y et al. *JAMA Psychiatry.*
